# Assessment of Physician Preferences for Large Language Model–Generated Responses Across Geographic Regions and Clinical Experience Levels: Preliminary Survey Study

**DOI:** 10.2196/82487

**Published:** 2026-01-27

**Authors:** James S Brooks, Paa-Kwesi Blankson, Peter Murphy Campbell, R Adams Cowley, Tsorng-Shyang Yang, Tijani Oseni, Anny Rodriguez, Muhammed Y Idris

**Affiliations:** 1 Morehouse School of Medicine Atlanta, GA United States; 2 Icahn School of Medicine at Mount Sinai New York, NY United States; 3 Department of Orthopedic Surgery MedStar Georgetown University Hospital Georgetown, DC United States; 4 Da’an District 280 Ren’ai Road, Section 4 Cathay General Hospital Taipei City Taiwan; 5 Department of Family Medicine, Edo University Iyamho Edo State Nigeria

**Keywords:** artificial intelligence, AI, large language model, physician, health communication, global health

## Abstract

**Background:**

Large language models (LLMs) have demonstrated increasing capabilities in generating clinically coherent and accurate responses to patient questions, in some cases outperforming physicians in terms of accuracy and empathy. However, little is known about how physicians across geographic regions and levels of clinical experience evaluate these artificial intelligence (AI)–generated responses compared to those authored by human clinicians.

**Objective:**

This study examined physician evaluations of LLM-generated versus physician-authored responses to real-world patient questions, comparing preference patterns across geographic regions and years in clinical practice.

**Methods:**

We conducted a cross-sectional online survey between March and May 2025 among licensed physicians recruited internationally. Participants reviewed anonymized medical responses from 2 LLMs (GPT-4.0 and Meta AI) and verified physicians to questions sourced from Reddit’s r/AskDocs forum. Each participant ranked 3 responses per question (1=most preferred; 3=least preferred) according to accuracy and responsiveness. Mean ranks, pairwise win proportions, and full rank distributions were analyzed descriptively and stratified by geographic region and years in practice.

**Results:**

Overall, LLM-generated responses were strongly preferred. GPT-4.0 achieved the best mean rank (1.63, SD 0.68; 95% CI 1.52-1.74), followed by Meta AI (1.83, SD 0.72; 95% CI 1.71-1.94), while verified physician-authored responses were least preferred (2.53, SD 0.76; 95% CI 2.40-2.65). In pairwise analyses, responses generated by GPT-4.0 won 78% (118/150) of the head-to-head comparisons versus physician-authored responses and 57% (86/150) versus Meta AI responses. Preference for GPT-4.0 was most pronounced in Africa (mean 1.59, SD 0.72), Asia (mean 1.91, SD 0.83), and North America (mean 1.55, SD 0.60), while Meta AI slightly led in Europe (mean 1.33, SD 0.57) and the Americas (mean 1.75). Across experience levels, physicians with less than 5 years in practice (28/52, 54%) ranked GPT-4.0 most favorably (mean 1.58, SD 0.63), followed by those with 10 to 15 years of experience (mean 1.56, SD 0.72). Even among physicians with more than 15 years in practice (9/52, 17%), AI-generated responses outperformed physician-authored responses (mean 1.75 vs 2.62). Across all subgroups, human-authored responses were ranked lowest.

**Conclusions:**

This exploratory study demonstrates that physicians across diverse regions and experience levels generally prefer LLM-generated responses to human-authored ones. The consistency of this finding across continents and practice durations underscores growing professional acceptance of AI as a viable tool for patient communication. These results suggest that modern LLMs, particularly GPT-4.0, may provide clinically acceptable, contextually relevant, and user-trusted health information, with potential to augment physician workflows and patient education.

## Introduction

The ability of artificial intelligence (AI) and large language models (LLMs) to analyze large volumes of health data and generate clinically accurate, context-aware responses presents a transformational opportunity to improve health care [1]. The adoption of LLMs in patient-facing clinical roles has progressed more slowly than in other areas, largely because of concerns about bias and diagnostic errors in previous models [2]. Methodological advancements, such as instruction tuning, retrieval-augmented generation, and multistep reasoning, have demonstrated significant improvements in both diagnostic accuracy and clinical utility of LLMs [3-5]. However, despite these advances, reactions within the medical community remain mixed. Surveys reveal a mixture of optimism and caution among physicians regarding the integration of LLMs into clinical and research practice [6,7].

Over the past 2 years, LLMs such as ChatGPT (OpenAI), Med-PaLM, and Claude have demonstrated increasing capability in performing complex clinical reasoning tasks, generating accurate discharge summaries, and offering patient-centered explanations comparable to those of human clinicians [8,9]. This progress has sparked active investigation into the safe and effective incorporation of generative AI into health communication workflows. Recent systematic reviews indicate that LLMs achieve high factual accuracy across common medical tasks but continue to exhibit variability depending on clinical specialty, input prompt quality, and language localization [10,11].

Understanding how physicians perceive the accuracy, appropriateness, and usefulness of LLM outputs is essential not only for assessing clinical accuracy but also for building trust in these models. Many studies have conducted physician evaluations of LLM-generated responses to patient questions, which play a crucial role in shaping broader perceptions and offer insight into both clinical utility and professional acceptance. Some studies have found that physicians rated LLM-generated responses more favorably than those authored by human clinicians [12,13]. However, previous research has been based primarily in North America and Europe, offering limited insight into how regional or experiential factors shape physicians’ evaluations of AI in clinical communication. Given that cultural context, health system infrastructure, and training experiences can influence technology acceptance, understanding global variation in physician attitudes toward AI is critical for equitable integration**.** In contrast, other studies, including those in specialized therapeutic areas such as rheumatology, have found that physicians rated LLM-generated responses more favorably than those authored by human clinicians [14]. These mixed results underscore the need for more refined evaluations of LLM performance in medical question-and-answer tasks.

As the capabilities of LLMs expand, their potential integration into clinical communication raises important questions about reliability, professional trust, and cross-cultural acceptance [15]. Although technical benchmarks and validation studies have demonstrated impressive gains in diagnostic reasoning and accuracy, these metrics alone do not capture how clinicians, those ultimately responsible for patient care, perceive the credibility and usefulness of AI-generated information [16]. Physician attitudes toward LLMs are likely shaped not only by individual experience but also by broader contextual factors, such as regional practice norms, health system maturity, and exposure to digital technologies [17,18]. Trust and adoption are influenced not only by technical performance but also by explainability, ethical transparency, and alignment with professional communication norms. For instance, studies have shown that even when LLMs outperform humans in accuracy, physicians may still perceive AI-generated responses as impersonal or lacking contextual depth [19]. Understanding these contextual dynamics is essential because perceptions of AI competence and safety directly influence the adoption and integration of AI into clinical workflows. Thus, beyond measuring algorithmic accuracy, evaluating how physicians from different regions and stages of clinical practice interpret and rank AI-generated medical responses provides a more nuanced view of the readiness of global medical communities to engage with generative AI in patient-facing roles.

This study extends existing research by comparing physician evaluations of LLM-generated versus human-authored medical responses across multiple geographic regions and levels of clinical experience. Specifically, we surveyed an international sample of physicians to assess anonymized responses from verified physicians and 2 general-purpose LLMs based on accuracy and responsiveness. By capturing perspectives from diverse settings, this work aims to identify how geographic and professional contexts influence physicians’ trust, preference, and acceptance of AI-generated communication in health care. Our preliminary findings demonstrate how contextual factors and professional experience shape evaluations of LLM-generated content, highlighting the need for larger, adequately powered studies to rigorously assess these differences.

## Methods

### Study Design and Participant Recruitment

We conducted a cross-sectional survey between March and May 2025. Eligible participants were licensed physicians and equivalent medical professionals recruited through institutional contacts, professional networks, and physician email distribution lists to ensure global representation. The recruitment email invited physicians to participate in a study comparing the accuracy and quality of responses to online medical consultations provided by physicians and by AI systems. Although the study topic was mentioned, invitations were distributed broadly and were not limited to networks or groups with a specific interest in digital health or AI.

No formal sample size calculation was performed because this exploratory study was designed to assess physician preferences rather than test a specific statistical hypothesis. The target sample size was determined pragmatically based on anticipated response rates from professional networks and similar physician survey studies, with the aim of obtaining at least 50 fully completed surveys to allow descriptive and subgroup analyses. Participants were asked to review and rank-order anonymized answers generated by 2 LLMs: GPT-4.0 (accessed via the ChatGPT Plus web interface [20]) and Meta AI.

Medical questions and physician-authored responses were sourced from Reddit’s r/AskDocs forum, one of the largest public repositories of health-related queries, with more than 689,000 members as of April 2025 [21]. This subreddit features responses from verified medical professionals, who are credentialed through moderator verification (eg, submission of a medical ID or a diploma). No custom or pretested prompts were developed for this study. As the original physician responses were written for patients within the Reddit forum, all model prompts replicated this patient-style question format verbatim to preserve contextual equivalence. Each medical question was copied verbatim from the Reddit r/AskDocs forum and entered directly into GPT-4.0 and Meta AI through their publicly available web interfaces without any editing, rephrasing, or contextual modification. Thus, both AI systems and physicians responded to the same patient-oriented questions, minimizing but not entirely eliminating task-framing differences. The exact question texts and corresponding model responses are provided in Multimedia Appendix 1.

A total of 86 question-answer pairs (from 300 of the most recent and highly rated physician-authored responses) were selected based on clarity, generalizability, and the absence of identifiable information. From this subset, 20 question-answer pairs were randomly selected using numbergenerator.org. All data collection procedures adhered to Reddit’s terms of service.

Finally, for each of the 20 selected medical question-answer pairs, new responses were generated using GPT-4.0 and Meta AI in isolated sessions to avoid contextual contamination. Consequently, each question had 3 responses: 1 from a verified physician, 1 from GPT-4.0, and 1 from Meta AI. No custom or pretested prompts were developed for this study. This approach preserved the authenticity and linguistic characteristics of real-world patient questions while ensuring that all model responses reflected genuine, unaltered interactions.

### Survey Content and Administration

The survey was constructed and administered using Qualtrics. Participants were recruited using a purposive sampling approach initiated through verified physicians within professional networks. Physicians were invited to complete the survey and share the invitation with other licensed colleagues who met the inclusion criteria. This approach facilitated recruitment of a diverse group of practicing physicians across multiple regions while maintaining respondent authenticity and professional verification.

Participants were asked to provide information about their primary specialty of practice, years in clinical practice, current level of practice, age, sex, and primary geographic region of practice. Participants were also asked to indicate their current reliance on AI in routine clinical practice. Each respondent was then presented with 3 anonymized responses (GPT-4.0, Meta AI, or a verified physician) to 3 randomly selected medical questions drawn from a bank of 30 and was asked to rank each based on perceived accuracy and responsiveness (1=most preferred; 3=least preferred). Specifically, respondents were presented with the following instruction:

For each of the following set of questions, you will be provided with 3 responses. Carefully consider the responses and rank (using the radio buttons on the right side of the answers) in order of which most appropriately answers the question: First button—most preferred; Second button—moderately preferred; Third button—least preferred There is no right or wrong order.

The order of the responses was randomized to reduce potential ordering bias, and all sources were anonymized to prevent identification.

### Statistical Analysis

Only complete survey responses were included in the analysis, and the survey design required all ranking and demographic questions to be answered before submission. Our primary outcome was the mean rank assigned to each system (1=most preferred; 3=least preferred) across all evaluation tasks. To examine subgroup variation, we compared mean ranks for each response type (ChatGPT, Meta AI, and verified physician) across categories of years in clinical practice and geographic region. To assess robustness, we conducted two sensitivity analyses: (1) the proportion of wins in pairwise comparisons between systems and (2) full rank distribution visualizations. Given the limited sample sizes within subgroups, no formal statistical comparisons were performed. Because the survey aimed to capture individual subjective preferences, no interrater reliability statistic was calculated. Rankings were treated as independent observations reflecting personal judgments rather than measures of rater agreement. All statistical analyses were conducted using R software (version 4.1.2; R Foundation for Statistical Computing).

### Ethical Considerations

This study was reviewed and approved by the institutional review board of the Morehouse School of Medicine (2276674-1) and determined to be exempt from full review because of its minimal risk, survey-based design. Participation was entirely voluntary, and informed consent was obtained electronically before respondents began the survey. All data were collected anonymously via Qualtrics, with no collection of IP addresses or other identifiable information, and were stored securely on access-restricted institutional servers. Results were analyzed in aggregate to ensure confidentiality. No monetary or nonmonetary compensation was provided.

## Results

A total of 52 physicians completed the survey, with nearly half (n=25, 48%) based in North America and the rest distributed primarily across Africa (n=13, 25%) and Asia (n=8, 15%). Most respondents were young, with more than half (n=28, 54%) aged between 25 and 34 years, and had less than 5 years of clinical experience (n=28, 54%). The sample was predominantly male (n=41, 79%). In terms of specialty, surgery was the most common (n=14, 27%), followed by family medicine (n=9, 17%) and anesthesiology (n=9, 17%). Other specialties were less represented (Table 1). On average, physicians reported integrating AI into approximately 19.1% (SD 19.6%) of their routine clinical practice, indicating varied but limited current adoption across the sample.

**Table 1:** Demographic and professional characteristics of physicians who participated in this global cross-sectional survey evaluating artificial intelligence–generated and physician-authored medical responses (N=52).

Variable			Participants, n (%)
**Age (y)**			
	25-34	28 (54)	
	35-44	14 (27)	
	45-54	5 (10)	
	55-64	4 (8)	
	≥65	1 (2)	
**Sex**			
	Male	41 (79)	
	Female	11 (21)	
**Specialty**			
	General	2 (4)	
	Surgery	14 (27)	
	Family medicine	9 (17)	
	Internal medicine	5 (10)	
	Anesthesiology	9 (17)	
	Emergency medicine	3 (6)	
	Pediatrics	2 (4)	
	Psychiatry	3 (6)	
	Other	5 (10)	
**Duration of clinical practice (y)**			
	10-15	6 (12)	
	5-10	9 (17)	
	<5	28 (54)	
	>15	9 (17)	
**Geographic region**			
	Africa	13 (25)	
	Americas	1 (2)	
	Asia	8 (15)	
	Asia-Pacific	3 (6)	
	Europe	1 (2)	
	North America	25 (48)	
	Other	1 (2)	

Table 2 displays the mean rank assigned to each response type across all physician evaluations, with lower scores indicating greater preference. Overall, responses generated by GPT-4.0 received the most favorable ratings, with a mean rank of 1.63 (SD 0.68; 95% CI 1.52-1.74). Meta AI responses followed, with a mean rank of 1.83 (SD 0.72; 95% CI 1.71-1.94), whereas verified physician-authored responses were ranked least favorably, with a mean of 2.53 (SD 0.76; 95% CI 2.40-2.65). These results suggest a clear overall preference among surveyed physicians for LLM-generated responses over those authored by verified physicians. In pairwise analyses, responses generated by GPT-4.0 won 78% (118/150) of the head-to-head comparisons versus physician-authored responses and 57% (86/150) versus Meta AI responses.

**Table 2:** Mean rankings of responses generated by GPT-4.0, Meta AI, and physicians by global physician respondents in a cross-sectional survey (n=150).

Response type	Rank, mean (SD)	SE	95% CI
ChatGPT-4.0	1.63 (0.68)	0.06	1.52-1.74
Meta AI	1.83 (0.72)	0.06	1.71-1.94
Physician	2.53 (0.76)	0.06	2.40-2.65

**Figure 1:**
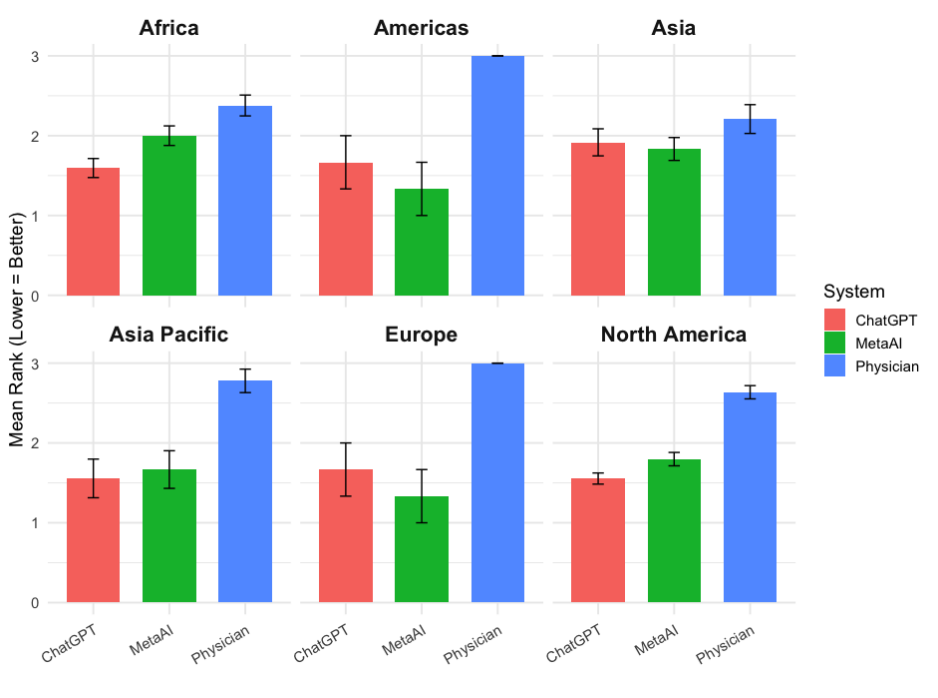
Mean ranking of GPT-4.0, Meta AI, and physician-authored medical responses by global geographic region among physician respondents.

Figure 1 presents the mean ranking of each response type, stratified by geographic region, along with associated error bars. Across all 6 regions, responses generated by GPT-4.0 and Meta AI were consistently ranked more favorably than physician-authored responses. Preference for GPT-4.0 was most pronounced in Africa (mean 1.59, SD 0.72), Asia (mean 1.91, SD 0.83), and North America (mean 1.55, SD 0.60), while Meta AI slightly led in Europe (mean 1.67, SD 0.57) and the Americas (mean 1.67, SD 0.57). In the Americas and Europe, Meta AI slightly outperformed GPT-4.0; however, both LLMs were clearly preferred over human-authored responses. Physician-authored responses received the highest (least preferred) rankings in every region, with particularly low ratings in the Americas and Europe. Although the overall preference for LLM-generated content was consistent, small regional differences in the relative performance of GPT-4.0 and Meta AI suggest some variation in how physicians across different regions evaluate AI-generated responses.

**Figure 2:**
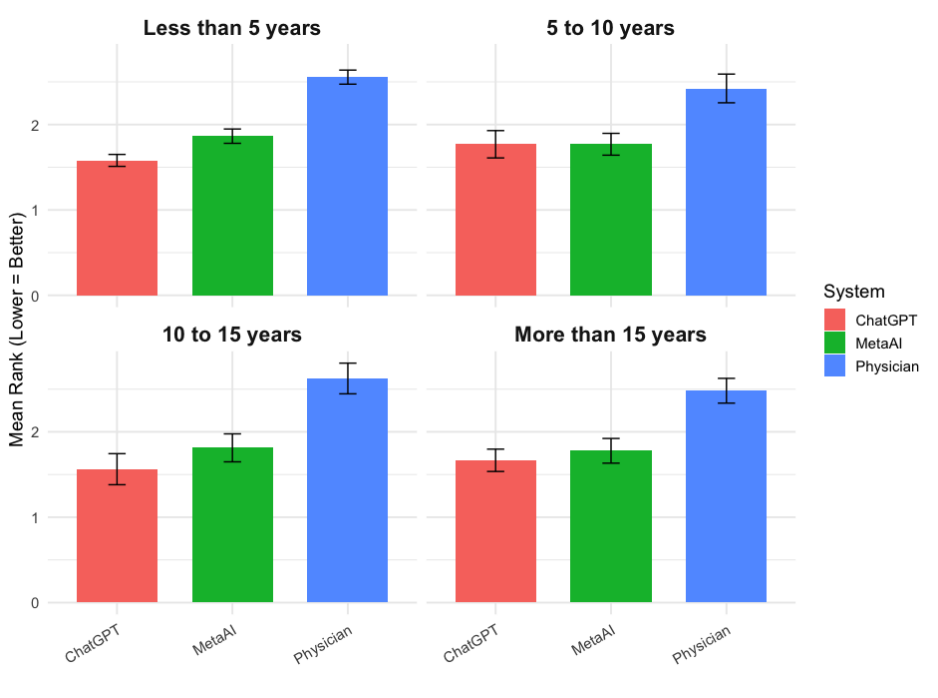
Mean ranking of GPT-4.0, Meta AI, and physician-authored medical responses by years of clinical practice among physician respondents worldwide.

Figure 2 displays the mean ranking of each response type stratified by years in clinical practice, with lower values indicating greater preference. Across all experience levels, physician-authored responses consistently received the least favorable rankings. Across experience levels, physicians with less than 5 years in practice (28/52, 54%) ranked GPT-4.0 most favorably (mean 1.58, SD 0.63), followed by those with 10 to 15 years of experience (mean 1.56, SD 0.72). Even among physicians with more than 15 years in practice (9/52, 17%), AI-generated responses outperformed physician-authored responses (mean 1.75 vs 2.62). Across all subgroups, human-authored responses were ranked lowest. Notably, the relative advantage of GPT-4.0 over Meta AI was most pronounced among those with 10 to 15 years of experience.

Results were consistent across sensitivity analyses. Pairwise comparisons confirmed the relative ordering of systems, and rank distributions revealed moderate variability across subgroups.

## Discussion

In this exploratory study, we demonstrated that LLM-generated responses were consistently preferred over those authored by verified human physicians. Overall, responses generated by GPT-4.0 received the most favorable mean rankings, followed by those generated by Meta AI, with physician-authored answers ranking lowest. This pattern held across geographic regions and years in clinical practice, with particularly strong preferences for GPT-4.0 in Africa, Asia Pacific, and North America, and for Meta AI in the Americas and Europe. Similarly, regardless of clinical experience level, both LLMs outperformed physician-authored responses, with GPT-4.0 maintaining a consistent advantage. Sensitivity analyses confirmed the robustness of these findings, supporting the overall conclusion that physicians generally favored LLM-generated medical responses.

The findings of this study both confirm and extend previous research on physician evaluations of LLM-generated versus physician-authored responses to medical questions. Consistent with existing work, we found that physicians generally preferred responses produced by LLMs such as GPT-4.0 and Meta AI over those authored by clinicians [12,13]. This trend was observed across all examined subgroups. However, not all previous studies found a clear preference for LLM-generated responses. Some studies have shown that ChatGPT's medical answers can be incomplete, inaccurate, or misleading when compared to clinician responses [14,22,23]. Others have found a significant performance gap in non–English language medical question answering, including an 18% drop in correctness, a 29% decrease in consistency, and a 13% decrease in verifiability, highlighting substantial limitations in the reliability and quality of LLM-generated health information across languages [24]. Altogether, the mixed results highlight the context-dependent and variable nature of LLM performance and physician evaluations.

The observed preference for LLM-generated responses across diverse regions and experience levels suggests that physicians increasingly perceive these systems as credible and clinically useful sources of information. This aligns with previous studies showing that ChatGPT and similar LLMs often produce responses rated higher in empathy, readability, and completeness than those authored by human clinicians, particularly in general medical advice contexts [8-10]. However, unlike previous studies conducted within single-country or specialty-specific samples, our findings extend this evidence globally, demonstrating that favorable perceptions of LLM-generated content are not confined to technologically advanced regions. The consistency of physician preferences across geographic and professional subgroups indicates a growing normalization of AI-generated communication within medical culture. At the same time, slightly higher rankings of LLM responses among early-career physicians may reflect greater exposure to digital technologies and openness to innovation, a pattern previously noted in studies of clinical AI adoption [11,12]. These results underscore the potential for LLMs to serve as scalable adjuncts for patient education, triage support, and clinician-patient communication, especially in settings with limited physician capacity. Nevertheless, while high favorability ratings suggest strong perceived accuracy and usefulness, they also emphasize the need for robust oversight, continuous validation, and clear governance frameworks to prevent overreliance on unverified AI outputs. Collectively, this study contributes new cross-regional evidence supporting the cautious but promising integration of generative AI into medical communication workflows.

The consistent preference for AI-generated responses may reflect their greater linguistic fluency, organization, and visual formatting compared to physician-authored replies, which were often less structured or more conversational in tone. Previous studies have shown that LLM outputs tend to emphasize empathy, completeness, and readability, characteristics that may enhance perceived quality even when clinical accuracy is comparable [15]. These stylistic and communicative advantages could make AI-generated responses more appealing to readers evaluating them for clarity and responsiveness, particularly in online, text-based contexts, when compared to real-world physician responses.

This study has several limitations that should be considered when interpreting the findings. First, the relatively small sample size limited statistical power, especially for subgroup comparisons by geographic regions and years in practice. As a result, observed differences in preferences should be interpreted as descriptive rather than inferential. This is especially true for physician-authored responses from Europe and the Americas, each of which included only 1 respondent. Second, the convenience sampling approach and voluntary participation may have introduced selection bias, as physicians with stronger opinions or greater familiarity with (and interest in) AI tools may have been more likely to respond. Third, although the use of real-world patient questions from Reddit’s r/AskDocs forum enhances ecological validity, the specific platform and format may not fully reflect the complexity of clinical communication in real-world practice nor are physician responses to questions on social media. Finally, the nonspecific construct of “preference” may have introduced variability in interpretation across respondents. Although rankings were analyzed as independent judgments, differences in how individual physicians conceptualized “preference” (eg, prioritizing accuracy versus clarity) could have contributed to interrater variability. Future studies with larger and more diverse samples should use mixed methods designs and incorporate standardized rating criteria or interrater reliability analyses to validate and extend these preliminary findings [12-14,22-24].

Although exploratory, this study contributes valuable evidence to the expanding literature, indicating that LLM-generated medical responses can be considered clinically acceptable by physicians. This suggests potential for LLMs to assist in patient education and clinical decision support when deployed under appropriate conditions. Our findings also underscore that physician perceptions of LLM-generated responses are not uniform but rather nuanced and influenced by various demographic factors (eg, geographic region) and professional characteristics (eg, years of clinical experience). These moderating factors may shape how clinicians evaluate the comprehensiveness, accuracy, readability, and overall usefulness of AI-generated outputs. Such variability highlights the importance of tailoring AI integration strategies to specific user groups and clinical contexts rather than assuming broad or universal acceptance. Therefore, while supporting the growing enthusiasm regarding the promise of LLMs, our study calls for continued, rigorous evaluation of AI systems across subgroups and by extension settings to ensure safe, effective, and responsible adoption.

## Appendix

Multimedia Appendix 1 Sampled patient questions obtained from the r/AskDocs forum and evaluated in this study.

## Abbreviations

AI: artificial intelligence
LLM: large language model

## Data Availability

The datasets generated or analyzed during this study are available from the corresponding author on reasonable request.
