# Supplementary material for: Assessment of Physician Preferences for Large Language Model–Generated Responses Across Geographic Regions and Clinical Experience Levels: Preliminary Survey Study
Source: JMIR Form Res. 2026 Jan 27;10:e82487. doi: 10.2196/82487 (PMC12892026; doi:10.2196/82487)
Supplement: Multimedia Appendix 1 [file formative_v10i1e82487_app1.pdf]

Assessment of Physician Preferences for Large Language Model–Generated Responses Across Geographic Regions and Clinical Experience Levels: A Preliminary Survey Study

SAMPLED QUESTIONS

Q9 For each of the following set of questions, you will be provided with 3 responses. Carefully consider the responses and rank (using the radio buttons on the right side of the answers) in order of which most appropriately answers the question:

- First button -**most preferred**
- Second button -**moderately preferred**
- Third button -**least preferred**

There is no right or wrong order.

End of Block: Block 1

Start of Block: Block 3

Q10 She is 37 F, 260 lbs, white, location USA, smokes, doesn't drink, some drug use (amphetamine) Questions about a family member displaying concerning behavior indicative of what I suspect could be a form of psychosis have arisen. These behaviors include erratic speech, slow response times, mentioning individuals from the past with whom she has negative associations, an inability to maintain focus during conversations, sudden outbursts of distress, expressing feelings of being unsafe, yelling not to be followed, and attempting to leave the premises whenever the thought arises. It's notable that she previously experienced a similar episode induced by methamphetamine in late 2019 to early 2020, which resulted in hospitalization for two weeks. Upon discharge, she was prescribed olanzapine at a dosage of 30 mg daily for approximately a year, after which she successfully tapered off and remained stable. However, she has intermittently used methamphetamine since then, albeit less frequently. Recently, she engaged in a prolonged binge of methamphetamine usage, leading to severe sleep deprivation and an escalation of symptoms within the span of about a week. Unlike the previous episode that manifested gradually over months, this onset was notably rapid. Given her previous hospitalization and the traumatic experience associated with it, there is understandable apprehension about seeking inpatient care again. There's also a question about the appropriateness of administering leftover olanzapine to alleviate her current symptoms and whether managing her condition as an outpatient is feasible. Additionally, navigating conversations and interactions with her poses challenges, particularly when attempting to prevent her from leaving, which elicits intense distress. Any insights or advice on managing this situation would be greatly appreciated.

- First button -**most preferred**
- Second button -**moderately preferred**
- Third button -**least preferred**

______ If you could calmly discuss that they are exhibiting signs of psychosis similar to the last time they used meth and you get no positive reaction in return, there isn't much you can personally do. Usually they wind up being a danger to themselves or someone else eg DUI, violence etc and then are involuntarily admitted. Does this person have a psychiatrist that they followed with because this might be a good person to reach out to for further guidance?I don't think giving

olanzapine without instructions is a good idea. This person needs not only help for psychosis but also substance abuse disorder as well. This can all be addressed outpatient but she is unlikely to have any self awareness or ability to have introspection in her current state thus making it difficult to get her into a physicians office. The ED can’t do much unless she has a life threatening situation eg heart attack from too much meth. Sorry this is happening. It’s always hard watching a family member suffer and you feel completely powerless to help. Substance abuse is a bear. (2)

______ It sounds like you are dealing with a very challenging situation, and your concerns are valid given her history and current behaviors. The rapid onset of psychotic symptoms following a prolonged binge of methamphetamine usage and severe sleep deprivation is concerning and suggests she may be experiencing a methamphetamine-induced psychotic episode. Given her history of a similar episode in the past, which required hospitalization and treatment with olanzapine, it is essential to approach this situation with caution. Ensuring her immediate safety is paramount. She should be in a safe environment where she cannot harm herself or others. If she is attempting to leave the premises or expressing intense distress, it might be necessary to have someone stay with her constantly. Although there is apprehension about seeking inpatient care, an evaluation by a healthcare professional is crucial. They can assess her current mental state, the severity of her symptoms, and the need for immediate intervention. Emergency services or a crisis intervention team may be needed if she becomes unmanageable or poses a risk to herself or others. Administering leftover olanzapine without a current prescription or medical advice is not recommended due to potential risks and the need for proper dosing and monitoring. A healthcare professional should evaluate whether restarting olanzapine or any other medication is appropriate. If inpatient care is not immediately necessary, a structured outpatient treatment plan involving regular check-ins with a psychiatrist, therapy, and support for substance abuse might be beneficial. Ensuring she has a supportive and stable environment will be crucial in managing her condition. When interacting with her, remain calm and reassuring. Avoid confrontations, and try to gently redirect her if she becomes agitated. Clear, simple communication can help her feel more secure. Addressing her intermittent methamphetamine use is essential for long-term stability. Engaging in substance use treatment programs, support groups, or counseling can help her maintain sobriety and prevent future episodes. Navigating this situation requires patience and support from family and healthcare professionals. Seeking advice from mental health professionals and substance use counselors can provide you with more tailored strategies and support. (5)

______ It appears that your family member may be experiencing symptoms of psychosis, which can be caused by various factors, including substance abuse. Methamphetamine use can trigger psychotic episodes, and the rapid onset of symptoms in this case suggests a possible link to her recent binge. Encouraging her to seek professional help from a medical provider or a substance abuse treatment center is crucial. A medical professional can assess her condition and provide guidance on appropriate treatment, which may include therapy, medication, or a combination of both. The National Alliance on Mental Illness (NAMI) Helpline (1-800-950-6264) and the Substance Abuse and Mental Health Services Administration (SAMHSA) National Helpline (1-800-662-HELP (4357)) are available resources for information and support. (6)

Q11 "How big of a deal is high blood pressure? I know the CDC says ""High blood pressure increases the risk for heart disease and stroke"" but I can't figure out if the part of a person's body

that causes high blood pressure also causes Heart disease and stroke or if high blood pressure literally causes heart disease. I'm 44F and I've been taking a medication for depression and anxiety off and on for over 15 years that causes a side effect of elevated blood pressure. For example, my normal blood pressure is like 104/70, but when take this medicine it tends to be more like 140/90. I'm getting more and more concerned about this as I get older. Especially since heart disease heavily runs in one side of my family. I stopped taking the medicine a few months ago because of those concerns; however, now I have a lower quality of life because my depression and anxiety are less regulated. (they weren't really fixed before but it was better than nothing) I know I could just start my medicine again and my depression and anxiety would get better but I also don't want to die when I'm like 50yo just to make things easier for me today. (My dad had a big heart attack at 50 and several since then). I have two young children I need to stick around for. I'm afraid of trying other possible medications for my depression and anxiety because I've had such a poor experience with them in the past. Do we know if High Blood Pressure CAUSES Heart Disease and stroke or if they are just correlated? Does it matter if the high blood pressure is caused by and outside source?" **1 (Most Preferred) 2 (Moderately Preferred) 3 (Least Preferred)**

______ To answer your question, high blood pressure causes heart disease through direct measures, it’s not a correlation. The first question is whether you actually have diagnosed high bp. That would be determined by seeing if your average bp (24 hours average) is more than 135/85 ish. You can have that tested with a 24 hour monitor through your doctors. If yes - then you should consider medication and lifestyle measures. You don’t say what medication it is that is causing your high blood pressure, but if it truly is causing it, that doesn’t mean you can’t take it. It means you should consider whether you could be more active, not smoke, eat less salt, work on stress levels, decrease caffeine, and lastly, consider taking a blood pressure med if none of those are helpful enough. It is very important to manage mental health as well and I wouldn’t recommend stopping a mental health medication for concern of high blood pressure without medical guidance recommending that. There are many anti depressants and anti anxiety meds that do not cause hypertension. Anxiety itself causes hypertension, as well. Consider talking all this through with your doctor. (2)

______ High blood pressure, or hypertension, is a significant health concern and is closely linked to heart disease and stroke. High blood pressure directly affects the cardiovascular system by causing the heart to work harder to pump blood. Over time, this added strain can lead to the thickening of the heart muscle, which can impair its ability to pump blood effectively, leading to heart disease. Elevated blood pressure can damage the inner lining of arteries, making them less elastic and more prone to plaque buildup (atherosclerosis). This can restrict blood flow to the heart and brain, increasing the risk of heart attacks and strokes. While high blood pressure is a major risk factor for heart disease and stroke, it is also often found alongside other risk factors such as high cholesterol, diabetes, and obesity. However, the evidence is strong that high blood pressure itself contributes to the development of these conditions. It’s not uncommon for medications, particularly those for mental health, to have side effects such as elevated blood pressure. This doesn't mean you have to accept this risk without alternatives. It might be worth discussing with your healthcare provider about potential adjustments in your medication or exploring other treatment options that do not significantly raise your blood pressure. Some antidepressants and anti-anxiety medications may have a lesser impact on blood pressure. Alongside medication, lifestyle changes such as diet, exercise, stress management, and smoking cessation can significantly help manage both depression and anxiety, as well as high blood pressure. Given your family history and personal medical history, it's crucial to regularly monitor your blood pressure and work with your healthcare provider to keep it within a healthy range. A holistic approach that includes medication, lifestyle changes, and possibly other therapies (e.g.,

cognitive-behavioral therapy, mindfulness, etc.) might offer a balanced way to manage both your mental health and cardiovascular risk. It's important to have an open and ongoing discussion with your healthcare provider about your concerns. They can help tailor a treatment plan that addresses both your mental health needs and cardiovascular risk. High blood pressure can directly contribute to heart disease and stroke. It's important to find a balance between managing your mental health and controlling blood pressure. There may be alternative medications or additional strategies that can help mitigate these risks. Regular check-ups and proactive management are essential, especially with a family history of heart disease. (3)
______ High Blood Pressure and Its Link to Heart Disease and Stroke High blood pressure (hypertension) is a significant public health concern, and for good reason. It is a major risk factor for heart disease and stroke, two leading causes of death and disability worldwide. The Connection: More Than Just Correlation High blood pressure is not just correlated with heart disease and stroke; it is a direct contributor to their development. Here's why: Damage to Blood Vessels: High blood pressure causes wear and tear on blood vessels, making them more susceptible to damage and plaque buildup (atherosclerosis). This can lead to heart attacks, strokes, and other cardiovascular events. Increased Cardiac Workload: High blood pressure forces the heart to work harder, which can lead to cardiac hypertrophy (enlargement) and decreased heart function over time. Kidney Strain: High blood pressure can damage kidneys, leading to chronic kidney disease and further increasing cardiovascular risk. Medication-Induced Hypertension In your case, the medication you're taking for depression and anxiety is causing elevated blood pressure as a side effect. This is a common concern, as many psychotropic medications can affect blood pressure. What You Can Do Discuss Alternative Medications: Talk to your doctor about exploring other medication options that may not have the same blood pressure-raising effects. Monitor Blood Pressure Closely: Regularly check your blood pressure, especially when starting or stopping medications. Lifestyle Changes: Adopt a healthy lifestyle, including a balanced diet, regular exercise, stress management, and adequate sleep, to help manage blood pressure and overall cardiovascular health. Consider Cardiovascular Risk Assessment: Ask your doctor about assessing your overall cardiovascular risk, including factors like family history, to determine the best course of action. Remember, it's essential to balance your mental health needs with your physical health concerns. Don't hesitate to discuss your concerns with your healthcare provider, and explore options that prioritize both your mental well-being and cardiovascular health. (6)

Q12 I'm not sure what to do but basically my mom (50F) is going in for surgery tomorrow morning, so her surgeon told her specific instructions to follow for the day before surgery (today). He told her to take 3 dulcolax pills at noon, which she did. Then she was supposed to mix an ENTIRE (238g) container of miralax with gatorade and drink 8 ounces every 15 minutes starting at 2:30pm until it was completely gone..she followed those instructions as well. Now shes been viciously vomiting for hours on end and I'm pretty sure she overdosed. I walked into her bathroom to hand her some water and it looked as if she was going to pass out at any second...she was severely pouring sweat but said she was freezing cold and said she felt like she was dying (genuinely). And it takes a lot for my mom to complain about something. What should we do?? Office hours to talk to her doctor are closed right now, is there anything we can actually do? She was also

instructed not to eat anything all day, so she's literally just throwing up all the gatorade and miralax. **1 (Most Preferred) 2 (Moderately Preferred) 3 (Least Preferred)**

______ I'd say she needs the emergency room as soon as possible for labs, an IV and imaging. She shouldn't be puking like that with a bowel prep and it's very concerning that she is. She may have a bowel obstruction if she can't keep anything down (if things can't pass, you throw them up). She's probably also severely dehydrated from the combination of the laxatives and throwing up. In addition to the dehydration, the electrolyte imbalance from diarrhea/vomiting can cause other issues. You are right to be concerned. I'm sorry this is all happening and I hope she gets better soon. (1)

______ I'm really sorry to hear about your mom's condition. Given the severity of her symptoms, it's crucial to act quickly. You should contact emergency services immediately by calling 911 or your local emergency number. Explain her symptoms, the medications she has taken, and the dosage. Until help arrives, if she can keep anything down, give her small sips of water or an electrolyte solution to help prevent dehydration, but do not force her to drink if she continues to vomit. Keep an eye on her vital signs such as breathing, heart rate, and consciousness, and inform the emergency responders immediately if she becomes unresponsive or has difficulty breathing. Additionally, prepare information for the paramedics by writing down all the medications she has taken, including the dosages and the times she took them. This will be useful information for the paramedics. Vomiting, sweating, and feeling cold can indicate severe dehydration and electrolyte imbalance, which can be life-threatening if not treated promptly. It's important to get professional medical help as soon as possible. (2)

______ Seek Immediate Medical Attention Your mother's symptoms, including severe vomiting, excessive sweating, and feeling like she's dying, indicate a serious situation that requires prompt medical attention. The combination of Dulcolax and Miralax may have caused an overdose of laxatives, leading to dehydration and electrolyte imbalance. Please Call Emergency Services or Go to the ER Call 911 or your local emergency number if your mother's condition worsens or she shows signs of severe dehydration, such as: Excessive thirst, Dark urine, Dizziness or fainting, Rapid heartbeat Visit the Emergency Room if you're unable to reach her doctor or if her condition doesn't improve. Contact the Surgeon's After-Hours Service (if available) to report the situation and seek guidance. Provide Hydration and Support Offer water or clear fluids in small amounts to help her stay hydrated. Monitor her vital signs, such as pulse, temperature, and blood pressure, if possible. Stay with her and provide emotional support. Delayed Surgery Considering her condition, it's likely that the surgery will be postponed until she's stabilized and cleared by her doctor. Keep an Eye on Her Condition. Continue to monitor your mother's condition closely, and seek immediate medical attention if her symptoms worsen or if you're concerned about her well-being. I hope this helps, and please let me know if there's anything else I can assist you with. (3)

Q13 We are at our children's hospital after a terrifying morning where she became weak and unresponsive after a bath. While we are still waiting for further blood test results, she tested positive for mono. I appreciate the measured responses from the physicians on this forum, but the replies from laypeople jumping to worst case scenarios are awful. Please remember that you are replying to real people with real concerns and fears, who are already reeling and anxious. My daughter is 5, almost 6, but very petite (in the 3rd percentile for growth). She had a mild fever

(99.5) beginning on Thursday afternoon, Friday it ramped up to 103. We went to the emergency room on Sunday as she suddenly became inconsolable and complained of neck pain. They tested her for flu, RSV, and COVID—all of which were negative. They told us to keep administering ibuprofen and Tylenol and just keep her hydrated. She was already small, but she hasn't eaten much for nearly a week and looks totally emaciated. She was seen by her PCP yesterday as the fever has not reduced at all, where they did a urinalysis (results all good) and she was tested for strep (negative). She suggested she may send her for blood tests if there's no improvement by today (there's not, her fever is 103.5 right now). I understand there isn't much treatment for a virus beyond what we're doing for her. Is there any utility in taking a terrified, weak 5 year old girl to a blood testing lab for something like this? I want my child to feel relief, I do not want to do something invasive and scary for little to no benefit. I am losing my mind right now. **1 (Most Preferred) 2 (Moderately Preferred) 3 (Least Preferred)**

______ I'm really sorry your little one is feeling unwell. If she does still have a fever on day 6, there is utility to doing some labs. We try to be very minimalist about labs in little ones, but more than 5 days of fever is a solid indication to look for more. There are certain inflammatory illnesses like Kawasaki disease that can present like that and so need to be tested for. It's much more likely to be a benign self limiting viral illness, but that's what I think her doctor is likely thinking. I'm not a pediatrician, but this is what I find pediatricians recommend when I call them from emergency about similar cases. (1)

______ I'm so sorry to hear about what your daughter and your family are going through. It's an incredibly stressful situation, especially when your child is so unwell and there aren't clear answers yet. It sounds like you're doing everything you can to get her the care she needs. Understanding Mono in Children: - **Mononucleosis (Mono)** is often caused by the Epstein-Barr virus (EBV) and can lead to prolonged symptoms, including high fever, extreme fatigue, sore throat, and swollen glands. While it's more common in teenagers and young adults, it can occur in young children as well, though the symptoms in children can sometimes be milder or present differently. Current Situation: - Given that your daughter tested positive for mono, it makes sense why she's experiencing these severe symptoms, including the persistent high fever, fatigue, and neck pain. - **Weakness and Unresponsiveness:** The weakness and unresponsiveness you observed, especially after a bath, are concerning, but it could be related to her body's extreme fatigue from fighting the virus. Blood Tests: - **Why Blood Tests?** Blood tests can help confirm the diagnosis of mono and assess her overall health, particularly if the illness has been severe or prolonged. They can check for signs of complications, such as liver function (mono can sometimes cause liver inflammation) or to rule out other conditions. - **Invasiveness vs. Benefit:** While blood tests are somewhat invasive, they can provide crucial information, especially in a child with such severe symptoms. Given that she's already in the hospital, it may be more reassuring to complete the tests now while she's under medical care, rather than waiting and possibly needing to return later. Comforting Your Child: - **Emotional Support:** Hospitals often have child life specialists who can help make the process of blood testing less scary for children by explaining the procedures in a child-friendly way, using distraction techniques, or providing comfort items like toys or videos during the test. - **Parental Reassurance:** Your presence and reassurance are key. Let her know you're there with her and that the tests are being done to help the doctors figure out how to make her feel better. Next Steps: - **Monitoring and Care:** Continue to monitor her closely, especially for any signs of worsening symptoms. Keep her hydrated and comfortable, and follow the doctors' advice on managing her fever and symptoms. - **Rest and Recovery:** Recovery from mono can take time, and it's important that she rests as much as possible. Encourage fluids and offer small, easy-to-eat foods if she's able to eat. Your Well-Being: - **Your Anxiety:** It's completely normal to feel anxious and overwhelmed in this situation. Try

to take care of yourself as well—reach out to family, friends, or even hospital support services for emotional support during this difficult time. You're doing everything right by advocating for your daughter's care and seeking medical advice when needed. Trust the medical team's guidance, and don't hesitate to ask them any questions you have about her treatment and recovery. Your daughter is in good hands. (2)

______ I'm so sorry to hear that your daughter is going through this. It's understandable that you're concerned and want to minimize her discomfort. Given her symptoms and positive mono test, it's likely that the blood tests will confirm the diagnosis. However, the tests may also help rule out other conditions that could be causing her symptoms. While it's true that viral infections often have limited treatment options, a definitive diagnosis can help guide her care and management. In some cases, medications like antivirals or steroids may be prescribed to help manage symptoms. Considering her age and vulnerability, it's reasonable to weigh the benefits against the potential distress of blood testing. You may want to discuss the following with her physician: The likelihood of the blood tests providing new information that would change her treatment plan The potential risks and benefits of the tests themselves. Alternative ways to monitor her condition and adjust her care without the need for blood tests. Remember, you are your daughter's advocate. It's essential to discuss your concerns and priorities with her healthcare team to determine the best approach for her care. (3)

Q14 16 - female - Caucasian - 103lbs - 5'8" - USA (New Hampshire) Born with thyroid issues and was taking a thyroid medication for many years, though I do not know if she still takes it. Let me start off by saying I am very close with my nieces and their mother (my sister) is one of my best friends. But, I think my sister is making all the wrong choices and my niece is going to end up dead. My niece "Emma" has been suffering from anorexia since September, though my sister and her husband kept it a secret from everyone else in the family (despite us being very close) until a few days before Christmas -- at which point my niece's doctors were recommending hospitalization given serious concerns regarding her blood pressure and the weakness of her heart. She has always been extremely thin and a very picky eater -- my sister and her husband allowed -- and has been very conscious of weight (other people's and her own) since a very young age: when she was 7, we were celebrating Father's Day (June in New Hampshire - it was in the high 70s/low 80s) and everyone was in shorts and tee shirts except Emma, who was wearing a sweatshirt and complaining she was cold. I jokingly told her that's because she has no body fat because she doesn't eat enough, and she responded "good, body fat is gross." When I asked her if she thought I was gross because I had body fat, she just kinda shrugged and was like "well...." Anyways, after Christmas Emma had to be withdrawn from school because her BMI was so low. She hasn't been allowed to drive (per DR's orders) since December. At Christmastime, doctors recommended she be put into a full-time residential program, but my sister and her husband were VERY resistant to this idea [she was previously hospitalized for an unrelated issue and was sexually abused by another patient] -- but after a few weeks, we were able to convince them to at LEAST do a partial hospitalization program (9-3 Monday thru Thursday, and on Fridays she's basically in a ZOOM version at home until the family goes to a family therapy session around dinner time that focuses on how the family can support her getting better). In February, my niece's PCP dropped her from care due to "legal" reasons because she wasn't getting better -- I'm guessing because my sister and her husband were not following medical advice. Since January,

my niece has only gained 8 pounds. Yesterday, the PHP told my sister that she needs to go into a full-time residency program; my sister and her husband are NOT on board and they think they can fix this themselves at home. According to my sister, "all a residency program will do is they will put food in front of her and tell her to eat, and if she refuses then they hospitalize her and put a tube up her nose." -- I don't believe that there is no therapy or anything else involved, as she claims. Also, I do NOT believe this is attention-seeking behavior -- she legitimately is afraid of food, she fights eating and she's also at the point where when we finally convince her to eat something, she feels sick (her teeth are black, which my sister claims is from so much Pepto Bismol use) and often vomits. She's also suffering from severe constipation and has had to go to the ER for enemas, etc. I am TERRIFIED my niece is going to die if my sister and her husband don't put her into residency, and am at a loss of what to do. My niece is a very smart, fun, beautiful, kind young girl who desperately needs help. I will say, that although I disagree with a lot of my sister and her husband's parenting choices, there is no doubt that they love their kids (this is not a child abuse situation) and are overwhelmed and struggling, but neither of them are fit/healthy/have good diets/get no exercise, and their education/expertise is in comp sci (software engineering) -- so it's not like they have the expertise to address this on their own (i mean, they tried from september until december and made no progress...) Is there ANYTHING i can do? Can anyone explain about Emma's PCP dropping her? Any suggestions for convincing my sister and her husband to follow doctor's advice? My sister keeps saying that this is just "so hard" -- even telling her its going to be harder to pick out Emma's casket has fallen on deaf ears. **1 (Most Preferred) 2 (Moderately Preferred) 3 (Least Preferred)**

______ This is certainly a hard situation, but your sister and brother-in-law don't seem to understand the gravity of Emma's medical situation. At this point, I'd be surprised if her medical team hadn't discussed having to make a call to child protective services. Unfortunately sometimes that's what it takes. Given that you have your own concerns about her safety in this situation, it may be worth you making a call as well. Calling doesn't mean that they're going to take custody away from her parents, but it makes sure that someone else is looking out for her health and safety where they may mandate that she get further treatment. (1)

______ It's evident that Emma's situation is dire and requires urgent intervention. Anorexia nervosa is a serious mental health condition that can have life-threatening consequences if not treated properly. The resistance to a full-time residential program from your sister and her husband is concerning, especially given Emma’s critical condition and the medical recommendations for more intensive care. Residential treatment programs for eating disorders involve much more than just monitoring food intake. They offer a structured environment with medical supervision, psychological therapy, nutritional counseling, and support from healthcare professionals trained to handle complex cases like Emma’s. The focus is not just on eating but also on addressing the underlying psychological issues contributing to the eating disorder. Emma’s PCP likely dropped her due to the complexity of her case and the lack of progress despite the partial hospitalization program. PCPs may refer patients to specialists or higher levels of care when their conditions become too complex or severe for routine management, particularly if the family is not fully complying with treatment recommendations. This is not uncommon in cases where specialized care is required. To address the situation, consider the following steps: Seek Professional Guidance by engaging with specialists in eating disorders or family therapy who can provide additional support and potentially mediate with your sister and her husband. Educate and Advocate by providing your sister and her husband with information about the benefits of residential treatment programs and the potential risks of not following through with recommended care. Sometimes, bringing in a medical expert to speak directly to them can make a significant impact. Involve Support Networks by mobilizing other family members or friends who may have

influence over your sister and her husband. A united, supportive approach can sometimes be more persuasive. Explore Emergency Options if you believe Emma's condition is life-threatening and immediate action is needed. You may consider contacting social services or child protective services to intervene, especially if you have evidence that her health is at serious risk. Continue to Offer Support by being a source of emotional support for Emma and encouraging her to engage with treatment, while also supporting your sister and her husband through their struggles. Your concern and efforts are crucial in ensuring that Emma gets the appropriate care she needs. It's a challenging situation, but persistent advocacy and seeking help from professionals can make a significant difference. (2)

______ I'm so sorry to hear that your niece is struggling with anorexia and that you're concerned about her well-being. It's understandable that you want to support her in getting the help she needs. Regarding the PCP dropping her from care, it's possible that the doctor felt that they could no longer provide the necessary care and support for Emma's complex needs. Sometimes, doctors may refer patients to specialists or more intensive programs if they require a higher level of care. As for convincing your sister and her husband to follow doctor's advice, it may be helpful to have an open and honest conversation with them about your concerns. You could express your love and support for Emma and your desire to see her get the help she needs. It may also be helpful to offer to help them research and find resources for residential programs or other forms of treatment. It's important to approach the conversation with empathy and understanding, rather than accusation or blame. You might say something like, "I know this is a difficult situation, and I'm worried about Emma's health. I want to support you and Emma in getting the help she needs. Can we work together to explore some options for treatment?" Additionally, you may want to consider reaching out to a mental health professional or a registered dietitian who specializes in eating disorders for guidance and support. They may be able to provide you with additional resources and information to help you navigate this situation. Remember that ultimately, you cannot force your sister and her husband to seek treatment for Emma, but you can continue to offer your love and support and encourage them to seek help. (3)

Q15 23F, I live in America, so this is why I'm hesitant. My dad just recently lost his job, so I have no health insurance. Im debating whether or not to just go. I really only want to go if this is life threatening. Before my shift yesterday I noticed I was exhausted, and my heart rate was pretty elevated (130's from just walking to the kitchen).During my shift I developed an awful headache. My mouth was extremely dry, and my upper back pain was severe. I felt like crap. I came home around 11 and took my temperature, it was 101.0 F. My headache got so bad it caused severe pain to move my eyes around. I woke up today around 6:30 am and called out of work, my fever was up to 102. I laid around all day with pretty bad aches and pains in my body, a serious headache, and shivering and shaking from being so cold.I took quite a few advil, but nothing My temp got to 103.5 at 10 pm, and 104.0 at 11:28. It's at 104.2 just now (11:45 pm). I took an at home covid test and it was negative. Im so scared. I've never had a fever so high in my life. I made the mistake of looking up my symptoms, and now I'm afraid I'm going to die if I don't go to the emergency room. I called to ask how much it would be and if I should come in, and the lady said she wasn't allowed to give any advice. What do i do? Update: just wanted to say thank you all again! I didn't really find anything out. I got a bunch of tests run on me, and nothing came back except for my white blood cell count and platelets being off. Waiting for blood cultures to come

back as well. Thank you to everyone for your advice and love during this really scary time ❤️ sending you all love Update 2: I got referred to an oncologist :(update 3: no cancer. Just a mystery! **1 (Most Preferred) 2 (Moderately Preferred) 3 (Least Preferred)**

______ ER immediately. Your symptoms strongly indicate a serious bacterial infection, possibly in the urinary or respiratory tract. The dry mouth and severe back pain could signify kidney involvement. (1)

______ I'm really sorry to hear that you've been going through such a scary and challenging experience. It's completely understandable to feel anxious, especially when facing a high fever and symptoms that don't improve with over-the-counter medication. Addressing Your Current Situation: 1. **High Fever and Symptoms:** - **Fever:** A fever of 104°F is very high and concerning, especially when combined with symptoms like severe headache, body aches, elevated heart rate, and shaking chills. - **Medical Attention:** It's good that you went to the emergency room, given the severity of your symptoms. High fever can sometimes indicate a serious infection or other medical condition that needs prompt treatment. 2. **Follow-Up on Tests:** - **White Blood Cell Count and Platelets:** An abnormal white blood cell count can indicate an infection, inflammation, or other issues in the body. The fact that these were off, along with your symptoms, warranted further investigation, which is why you were referred to an oncologist. - **Blood Cultures:** Waiting for blood culture results is important as they can help identify if there's an infection in your blood, such as sepsis, which could be causing your symptoms. 3. **Oncology Referral:** - **Referral to an Oncologist:** While being referred to an oncologist is certainly scary, it's a step to ensure that all possible causes, including those related to blood disorders or cancers, are thoroughly investigated. It's good news that the results came back negative for cancer, but it's still important to continue following up to find the root cause of your symptoms. Next Steps: 1. **Stay in Touch with Your Healthcare Providers:** - **Follow-Up:** Ensure that you follow up on all test results and attend any recommended appointments. Even though the initial tests didn't point to a clear diagnosis, your symptoms need to be closely monitored. - **Monitor Symptoms:** Keep a log of your symptoms, including fever, heart rate, and any new developments. This can help your healthcare providers track your progress and adjust treatment as needed. 2. **Managing Costs:** - **Financial Concerns:** Without insurance, medical bills can be overwhelming. However, many hospitals offer financial assistance programs or payment plans. Don't hesitate to ask about these options. - **Health Clinics:** If further follow-up is needed, consider visiting a community health clinic or low-cost healthcare provider if available in your area. 3. **Health Maintenance:** - **Stay Hydrated:** High fever can cause dehydration, so it's important to drink plenty of fluids. - **Rest:** Allow your body to rest and recover as much as possible. Avoid strenuous activities until you're feeling better. Conclusion: The most important thing is that you sought medical attention when your symptoms were severe. Now that you've ruled out some serious conditions, it's crucial to keep working with your healthcare providers to figure out what's going on. Even though this is a frightening experience, you're doing the right things by staying informed and proactive about your health. Please take care, and don't hesitate to reach out to your healthcare team if you have any new symptoms or concerns. (2)

______ I'm not a doctor, but I can try to provide some general guidance. Based on your symptoms, it's understandable that you're concerned and seeking advice. A fever above 103°F (39.4°C) can be a cause for concern, especially if it's accompanied by other severe symptoms like a headache, body aches, and shivering. Please seek medical attention immediately. While I understand your concerns about not having health insurance, many hospitals are required to treat emergency conditions regardless of insurance status. Delaying medical attention could lead to worse

outcomes. Go to the emergency room or urgent care. Don't hesitate to seek help. A medical professional can evaluate your symptoms and provide a proper diagnosis and treatment plan. Remember, it's always better to err on the side of caution when it comes to your health. I hope this helps, and I wish you a speedy recovery! (3)

Q16 26F, 5'7", 190lbs, Caucasian, extensive list below, some symptoms 2yrs but significantly worsened in the past two weeks, pcos/endometriosis/vitamin d deficiency/moderate sleep apnea, concerta 27mg extended release/vitamin D2 50,000 IU, no drinking/smoking/recreational drugs. Hoping I can get some insight from another neurologist. Having signs of dementia at only 26, and worried I've been seeing warning signs of it the past two years but passed it off as not having been medicated for my ADHD at the time. Deja vu, cognitive decline, increasing confusion, reduced concentration, feeling withdrawn because my ability to do usual things has been getting worse, feeling like my surroundings are unfamiliar, I can't tell if I'm psyching myself out or if I'm struggling with faces, feeling the need to repeat myself in conversation, and I'm constantly blanking and getting irritated and am hesitant to speak bc I keep having trouble word pulling. I feel less coordinated, too. I've noticed the past few years my vison has gotten worse and I see halos, too. I tested myself on the SLUMS and MoCa tests yesterday; scored 19-20 and 26. Dementia level on SLUMS and the lowest possible for normal on MoCa. I'm terrified. I'm afraid I've gone past the point of early diagnosis because myself and my neuro wrote it off as something benign. Talked to my previous neuro about my deja vu and trouble thinking, but she suspected sleep deprivation from the sleep apnea. She suggested an EEG even though she didn't suspect seizures. Didn't suspect the chance of dementia when I brought it up. My previous neuro is retiring and I spoke to my new neuro yesterday, and he seemed to just completely brush off my concerns. I tried to push for a spinal tap to see if I could get some answers. I actually wanted a spinal tap years ago when I was exhibiting unexplained neurological symptoms that seemed to correlate to MS, but things eventually subsided so I left it alone. New neuro wouldn't budge, saying that because my past two MRI's in 2020 and 2021 have been clear that we didn't need to order that. I asked if there were any tests at all that we could do that ruled out alzheimers or dementia and he seemed baffled and replied with "20 year olds don't get it." I'm already afraid of not being taken seriously by this person. I know the chances are so incredibly low being 26, but my symptoms keep telling me otherwise. I want to improve my lifestyle but I feel the need to focus on ruling out this first and foremost. If that's what's going on, I want to get treatment for it asap. Focusing now on getting a new neurologist and a second opinion. I feel like something is very wrong. History: Great grandmother, grandmother, and uncle on mom's side all have had/have alzheimers or dementia. Great grandmother and grandmother were over 60+ and my uncle who just got recently diagnosed is 65. My mother might be exhibiting signs and she's 65+. My uncle is a war veteran, has drank heavily in the past, has had head injuries, and did drugs. My grandmother had unmanaged diabetes. I had a minor concussion around in 2014 from falling off my horse. I have combined ADHD, OCD, PTSD, depression, and anxiety. I've been taking Concerta Extended Release 27mg for going on three months, and 50,000IU of Vitamin D2 for about four months. I'm very sedentary and have stayed inside constantly since the pandemic due to derealization when I go out the past two years. I almost never leave my room. I had a CPAP for a few months but didn't use it enough for my insurance to want to keep paying for it(because I had to switch to a different mask, therefore I wasn't using it for a few weeks until I got a new one.) I caught COVID back in October after a trip across the US. Up until recently, it's been easy to go into coughing fits

from my lungs being irritated, but it's been getting better. UPDATE: I haven't improved at all since this post, unfortunately. I've only gotten worse, it seems. I've made an appointment with my primary for the 1st of May to get bloodwork done that's required for being referred to a neuro in my area that specializes in neurodegenerative disorders. I'm going to try and get a CT scan tomorrow, and hope and pray that there's no atrophy and that this is all just something reversible.
1 (Most Preferred) 2 (Moderately Preferred) 3 (Least Preferred)

______ An ordinary neurologist is not equipped to diagnose or treat questionable dementia symptoms. For such cases a neuropsychiatrist affiliated to an academic center should be consulted. There are a multitude of tests, both psychiatric and neurological, that may be needed to diagnose your problem. Please consult a more qualified individual than just some random neurologist. (1)

______ Given your symptoms and concerns, it's understandable that you're feeling anxious and frustrated. Your family history of Alzheimer's and dementia, coupled with your current cognitive symptoms, certainly warrants thorough investigation, even though dementia is uncommon in someone as young as 26. Your symptoms are particularly concerning given your family history and recent cognitive decline. Although early-onset dementia is rare, it's crucial that your new neurologist takes your concerns seriously. A thorough neurological examination, including cognitive testing beyond SLUMS and MoCA, is essential. You might consider requesting neuropsychological testing, which provides a more detailed evaluation of cognitive function and may offer insights into specific areas of decline. While your MRIs from 2020 and 2021 were clear, a new MRI or CT scan could be valuable, especially if your symptoms have worsened since those scans. A more recent imaging study might reveal changes that were not present before. Discuss with your neurologist the possibility of advanced imaging techniques, such as functional MRI (fMRI) or PET scans, which could offer more detailed insights into brain function and structure. Several medical conditions can mimic or contribute to cognitive decline, including vitamin deficiencies, thyroid dysfunction, and other metabolic issues. Given your vitamin D deficiency, it might be worth checking other vitamin levels (such as B12), as well as thyroid function and other markers of metabolic health. Sleep apnea, which you've been diagnosed with, can have a significant impact on cognitive function, and untreated sleep apnea is associated with an increased risk of cognitive decline. It's crucial that you address this, perhaps by trying to restart CPAP therapy with a mask that works for you. Ensuring good sleep hygiene and treatment adherence might alleviate some of your symptoms. Your psychiatric conditions, including depression, anxiety, PTSD, and OCD, can also contribute to cognitive symptoms. A psychiatrist or psychologist might help differentiate between cognitive symptoms caused by these conditions and those due to other neurological issues. It's positive that you're seeking a second opinion, especially with a neurologist specializing in neurodegenerative disorders. Ensure you communicate your symptoms clearly, your concerns about your family history, and your desire for comprehensive testing. Sometimes, bringing a family member or friend to appointments can help ensure your concerns are fully heard and addressed. Although it's essential to rule out serious conditions first, improving your lifestyle can have significant positive effects on cognitive health. Regular physical activity, social engagement, and a balanced diet rich in nutrients that support brain health (such as omega-3 fatty acids) can all be beneficial. Given your history of COVID-19 and ongoing symptoms, it might also be worth discussing the possibility of long COVID with your healthcare provider, as some people experience prolonged cognitive symptoms after recovering from COVID-19, which could potentially explain some of your issues. Finally, it's important to take care of your mental health while navigating these medical concerns. The uncertainty and stress

you’re feeling are completely understandable, but try to focus on the steps you’re taking to seek answers and advocate for your health. (2)

______ Neurological Concerns and Seeking a Second Opinion You're a 26-year-old woman with a history of PCOS, endometriosis, vitamin D deficiency, and moderate sleep apnea. You're experiencing symptoms that are concerning you, such as: Déjà vu, Cognitive decline, Confusion, Reduced concentration, Feeling withdrawn, Unfamiliar surroundings, Difficulty with face recognition, Repeating yourself in conversations, Word-finding difficulties, Coordination issues, Vision problems (halos). You've taken the SLUMS and MoCa tests, scoring 19-20 and 26, respectively, indicating possible dementia. Your previous neurologist dismissed your concerns, and your new neurologist seems unresponsive. You're seeking a second opinion and want to rule out Alzheimer's or dementia. Family History and Medical Background You have a family history of Alzheimer's and dementia on your mother's side. You've experienced a minor concussion in the past and have been diagnosed with combined ADHD, OCD, PTSD, depression, and anxiety. You're currently taking Concerta Extended Release 27mg and Vitamin D2 50,000 IU. Next Steps You're scheduled to see your primary care physician on May 1st for bloodwork and a potential referral to a neurologist specializing in neurodegenerative disorders. You're also seeking a CT scan to rule out atrophy. Recommendations Seek a second opinion: Find a neurologist who will listen to your concerns and take them seriously. Get a thorough evaluation: Push for a comprehensive workup, including imaging studies (e.g., MRI, CT scans) and cognitive tests. Consider a specialist: Look into neurologists specializing in neurodegenerative disorders, such as dementia or Alzheimer's. Keep a symptom journal: Track your symptoms and any changes to share with your healthcare providers. Stay proactive: Don't give up until you find a doctor who takes your concerns seriously and helps you get the answers you need.Remember, it's essential to advocate for yourself and your health. Keep pushing forward, and I hope you find the answers and support you need. (3)

Q17 My Girlfriend (26F 180 lbs) and I (25M 200 lbs) Recently had a daughter who was born this passed Friday and it was an absolutely beautiful experience. It was amazing and the feeling that I felt in that moment was something that I have never felt in my entire life. Pure happiness. Not even maybe 5 minutes after the daughter was placed on my girlfriends chest, the nurse practitioner approached us and let us know that our daughter will have to be placed in the NICU due to one of my girlfriends blood screenings returning positive for Syphilis. Apparently on Tuesday when we had a false labor hospital visit they took her blood for labs and her RPR screening showed positive for syphilis antibodies. They retested her on Wednesday at our weekly appointment and the results tested positive as well. She had these same tests done at 11 and 28 weeks and both screens showed no signs of syphilis. Our daughter was immediately started on penicillin and she gets her dose via IV every 12 hours. A few days later the blood titer results for my girlfriend and daughter came back and showed my daughter being 1:1 and my girlfriend being 1:16. I am still in the process of getting my test results in. With all of this being said I guess my questions are the following: -How is it possible the RPR screens at 11 and 28 weeks showed nothing alarming but they were detected 3 days prior to my daughters birth? I can very confidently say that I have never cheated on my girlfriend and I was wondering if I should be worried something happened with her and another person from that 28 week mark up until now. I am very confident she would never do anything like that but I just have no other explanation at this point.

-what do the 1:1 and 1:16 titer results mean exactly? Are those good signs or bad signs? -can the RPR syph screening produce a false negative or a false positive on two separate occasions? -Is my daughter at risk for any long term health issues due to her condition. -In the case that one of us have had syphilis for years without knowing how long would the time frame be to where we would have to worry about serious complications in our health? Neurosyphilis really freaks me out especially. Anything will help at this point. We are both emotionally drained and going down the Google rabbit hole has not been helping. Thank you so much. **1 (Most Preferred) 2 (Moderately Preferred) 3 (Least Preferred)**

______ Syphilis testing requires two tests: an RPR and a syphilis specific test. Did your girlfriend get the syphilis specific test too? Without it you can't diagnose syphilis. It's possible the nurse practitioner doesn't realize that and jumped the gun. RPR can be elevated for lots of things other than syphilis, including pregnancy itself! (1)

______ First of all, congratulations on the birth of your daughter! It sounds like you've been through a lot, and it's completely understandable that you're feeling overwhelmed and confused by everything that's happening. I'll do my best to address your concerns one by one. 1. Why did the RPR screens at 11 and 28 weeks show nothing alarming, but they were detected just before birth? The RPR (Rapid Plasma Reagin) test is a screening tool used to detect syphilis. It measures the presence of antibodies that your body produces in response to the bacterium that causes syphilis. There are a few possibilities for why the tests earlier in the pregnancy were negative and then became positive: False Negatives: It's possible that the earlier tests were false negatives, meaning that the infection was present but not detected. This can happen if the infection was in a very early stage or if there were very low levels of antibodies at the time of testing. Recent Infection: It's also possible that the infection was acquired after the 28-week test. However, you mentioned that you and your girlfriend are confident that there has been no infidelity, so this might not be the case. Fluctuating Antibody Levels: In some cases, the levels of antibodies can fluctuate, leading to changes in test results over time. However, this is less common. 2. What do the 1:1 and 1:16 titer results mean? The titers refer to the concentration of antibodies in the blood. A titer of 1:16 in your girlfriend means that the antibodies were detected at a dilution of 1 part blood to 16 parts diluent. A higher titer (like 1:16) generally indicates a more recent or active infection, while a lower titer (like 1:1) may indicate an older or less active infection. Your daughter's titer being 1:1 suggests a lower level of antibodies, which is often seen in congenital syphilis (when a baby acquires syphilis from the mother during pregnancy). 3. Can the RPR syph screening produce a false negative or a false positive on two separate occasions? Yes, the RPR test can produce both false negatives and false positives. Factors like recent infections, autoimmune diseases, pregnancy itself, or even improper handling of the sample can lead to inaccurate results. False positives are relatively common with RPR, which is why a confirmatory test (like the FTA-ABS or TP-PA test) is often done if there's a positive RPR result. 4. Is your daughter at risk for any long-term health issues due to her condition? Congenital syphilis can have serious long-term effects if left untreated, including developmental delays, organ damage, and neurological issues. However, since your daughter has been started on penicillin treatment right away, this greatly reduces the risk of long-term complications. It's crucial to follow up with her healthcare provider to monitor her health and development. 5. In the case that one of you has had syphilis for years without knowing, how long would the time frame be to where you would have to worry about serious complications in your health? Syphilis progresses in stages (primary, secondary, latent, and tertiary). If left untreated for years, syphilis can progress to the tertiary stage, which can cause serious complications such as neurosyphilis, cardiovascular problems, and damage to other organs. Neurosyphilis: can occur at any stage of syphilis, but it is more common in the later stages. If someone has had untreated syphilis for several years, they could be at risk for these

complications, but this varies from person to person. If either of you had undiagnosed syphilis for a long time, treatment with antibiotics (like penicillin) is very effective in preventing progression and complications, even in later stages. 6. Diagnostic Tests Coming Back Negative: It's excellent news that the confirmatory tests came back negative, which suggests that the initial RPR result might have been a false positive. This is more common than people realize, and it means that the concern about syphilis might have been a false alarm. It's great that you are going home and that things are looking up. Continue to monitor your daughter’s health closely and stay in touch with your healthcare providers for follow-up care. It's understandable to feel emotionally drained, but it sounds like you're getting good care, and things are moving in the right direction. If you have any more questions or need further clarification, feel free to ask. (2)

______ Congratulations on the birth of your daughter! I'm so sorry to hear that this joyful moment was followed by concerns about syphilis. I'll do my best to address your questions and concerns: Timing of syphilis detection: It's possible that your girlfriend contracted syphilis between 28 weeks of pregnancy and the pre-delivery screening. Syphilis can remain undetected for a period, and the RPR screen may not have shown antibodies yet at 28 weeks. Titer results: The 1:1 and 1:16 titer results indicate the level of syphilis antibodies present. A higher titer (1:16) indicates a more active infection, while a lower titer (1:1) suggests a less active or potentially treated infection. In your daughter's case, the 1:1 titer is a good sign, indicating that the treatment is working. False negatives and false positives: Yes, RPR screens can produce false negatives (early in the infection or if the test is taken before antibodies develop) or false positives (due to other medical conditions or infections). However, a positive result on two separate occasions is unlikely to be a false positive. Long-term health risks: If left untreated, syphilis can lead to serious complications, including neurosyphilis. However, with prompt treatment, the risk of long-term health issues for your daughter is significantly reduced. Duration of untreated syphilis: If you or your girlfriend have had untreated syphilis for years, the risk of serious complications increases. Neurosyphilis can occur 10-30 years after initial infection if left untreated. (3)

Q18 I’m a 36 year old Female weight 126lb height 5’5 I don’t drink/ smoke or take medication. I was diagnosed with pink eye and I am taking anti bacterial eye drops. Will red light therapy from the gym help with the swelling? I’m traveling next week and want it gone. Any suggestions would be appreciated **1 (Most Preferred) 2 (Moderately Preferred) 3 (Least Preferred)**

______ Don't shine a light in your eye. Won't help and may damage your eye (1)

______ Red light therapy has been explored for various health benefits, including reducing inflammation and promoting healing. While it might offer some relief for inflammation, it's essential to approach it cautiously, especially around the eyes. If considering red light therapy, ensure the device is specifically designed for use near the eyes and use protective eyewear if required. It is crucial to consult with your healthcare provider before starting red light therapy to ensure it's safe

for your specific condition and situation. Use the therapy in moderation as recommended by the manufacturer or your healthcare provider. (2)

______ While red light therapy has anti-inflammatory properties, its effectiveness for pink eye (conjunctivitis) is largely anecdotal. There's limited scientific evidence to support its use for this condition. Instead, Focus on Proven Remedies To reduce swelling and promote healing, consider: Applying a warm compress to the affected eye.Keeping the eye clean and avoiding irritants. Continuing your antibacterial eye drops as prescribed. Getting plenty of rest Additional Tips for Travel. Wear sunglasses to protect your eye from wind and dust Avoid sharing makeup or cosmetics. Keep your hands clean and avoid touching your eyes Consult a Healthcare Professional If your symptoms worsen or don't improve with treatment, consult your healthcare provider or an eye specialist (ophthalmologist) for further guidance. Hope this helps, and have a safe trip! Click to write Item 3 (3)

Q19 37 male heavier set but not over weight and only history is anxiety meds. No heart issues in the past or blood pressure issues. Woke up with left tingly arm and went to coach his daughters soccer team. Today at the soccer field around 9 his chest hurt and passed out. Woke up and was okay just chest pain. A bystander called 911. When the paramedics arrived they hooked him up the EKG and said he was having an active heart attack. They left for the hospital immediately and the wife had someone drive her. They advised her to not ride in the ambulance as they were going to be driving very fast and she was crying and screaming naturally. When they got to the hospital he passed out again and heart rate was 30. He woke up and his memory is gone. Didn’t know he was at soccer and if his wife was there after he had seen her. Didn’t remember telling to his daughter on the phone. Now blood work is saying no heart attack and they think he has a blockage in his brain. As the day continues his memory gets worse. He’s been admitted for observation and more testing. They kept saying it’s anxiety because he takes anxiety meds but his wife said no something is wrong. Then he had to go to the bathroom and they un hooked him and let him go in alone and he passed out again. Any thoughts? Edit to add- they have him scheduled for a stress test tomorrow. And also if it is a blockage, will his memory come back once fixed? **1 (Most Preferred) 2 (Moderately Preferred) 3 (Least Preferred)**

______ My thoughts are that since he is being admitted the docs can sort out if anything dangerous is happening. Two or three negative troponins... and he 100% isn't having a heart attack (even two usually gets you there). They should pretty easily be able to evaluate for stroke with CTA and MRI (if they are actually worried... as a bystander with this info this story doesn't make me think stroke). Now... what cannot be tested for? Anxiety, pseudo seizures, and "falling out" due to anxiety. We can rule out bad stuff though! Stress test is pretty great considering they probably have 2 negative trops. He's being taken very seriously just in case his wife doesn't think so. (1)

______ Given the symptoms described—tingling in the arm, chest pain, passing out, memory loss, and the initial misdiagnosis of a heart attack—it sounds like your friend may be experiencing a significant medical issue that could involve either cardiac or neurological problems, or potentially both. The fact that his memory is deteriorating and he is passing out repeatedly suggests a serious

condition that might not solely be related to anxiety. While anxiety can cause physical symptoms, such as chest pain and fainting, the severity of his symptoms and the acute changes in his mental status are concerning. The discrepancy between the initial diagnosis of a heart attack and subsequent tests indicating no heart attack might indicate a complex case where multiple issues are at play. A blockage in the brain, possibly a stroke or transient ischemic attack (TIA), could explain the symptoms of sudden memory loss and fainting. Strokes or TIAs can cause temporary or permanent changes in brain function, including memory issues, depending on the location and severity of the blockage. The stress test scheduled for tomorrow will provide additional information about his heart's condition and how well it responds to exertion. Meanwhile, continued observation and more diagnostic tests, such as imaging of the brain and further cardiovascular assessments, will be critical in determining the exact cause of his symptoms. If it is a blockage or stroke, treatment and recovery depend on the extent and location of the blockage. In many cases, if the blockage is identified and treated promptly, memory and other functions can improve significantly. However, recovery can vary depending on the severity of the brain damage, if any. In summary, it's important for his medical team to thoroughly evaluate both his cardiac and neurological health to determine the underlying issue. Close monitoring, further diagnostic tests, and appropriate treatments are essential to addressing his condition and hopefully restoring his memory and overall health. (2)

______ Possible Causes and Concerns Based on the symptoms described, it's understandable that the medical team is considering various possibilities, including anxiety and a potential blockage in the brain. The patient's history of anxiety medication and the absence of heart issues or blood pressure problems in the past suggest that cardiovascular issues might not be the primary concern. However, the EKG results indicating an active heart attack and the subsequent chest pain and loss of consciousness require further investigation. Memory Loss and Blockage The patient's worsening memory loss and the suspicion of a blockage in the brain are concerning. A blockage in the brain could be related to various conditions, such as a transient ischemic attack (TIA or "mini-stroke"), a stroke, or a vascular issue. If a blockage is confirmed, it's possible that resolving the issue could lead to improved memory function. However, this depends on the severity and location of the blockage, as well as the extent of any potential brain damage. Stress Test and Further Testing The scheduled stress test will help determine if there are any underlying cardiovascular issues that may be contributing to the symptoms. Additionally, further testing, such as imaging studies (e.g., CT or MRI scans), may be necessary to investigate potential blockages or other brain-related issues. Advocacy and Collaboration It's essential for the patient's wife to continue advocating for her husband's care and to collaborate with the medical team to ensure that all possible causes are explored. Encourage the medical team to consider multiple perspectives and to continue monitoring the patient's condition closely. Seeking Specialist Input Given the complex and evolving nature of the patient's symptoms, it may be beneficial to consult with specialists, such as a neurologist or a cardiologist, to provide additional expertise and guidance. Please keep me updated on the patient's progress and any new developments or test results. I'm here to support you both. (3)

Q20 Hello I'm heart broken I need to know if my dad felt pain or scared. My dad suddenly died at home yesterday, we think heart attack that led to cardiac arrested in his sleep apparently. I'm heart broken I can't eat or sleep, I keep seeing his face everytime I close my eyes, I wake up gasping for air. I've never seen a dead person before, I went to see his body yesterday and police

were there, he was lying on the floor stone cold and no colour to his face, I wish I didn't go and see him like that, it's haunting me and traumatised me, I loved him so much I want him back I'm hurting and I want to lash out, everytime I go to eat I think of his body on the floor and I can't eat yet my belly is so hungry, I can't sleep I don't know what to do…I have struggled with my mental health my whole life and I have CPTSD and I was slowly healing through EMDR and now I feel like this is a blow…I don't want him to have suffered. Would he of felt pain? Scared? He was alone? I want him to wake up,,,I don't feel like I'm in reality right now, my eyes feel heavy, It keeps dawning on me my beautiful kind dad is gone and never coming back, he lying in the morgue all alone I want to be lying next to him I want to be with him…the man I seen yesterday all sunken cheeks and Grey skin and cold didn't look like my dad it's haunting me when I close my eyes…I need to know hes at peace…I don't want him to have felt pain and scared? Please help I don't know we're to turn too I want him back **1 (Most Preferred) 2 (Moderately Preferred) 3 (Least Preferred)**

______ Dying in your sleep is an amazing and peaceful way to go. I'm sorry OP. It's normal to feel the way you do right now. (1)

______ I'm deeply sorry for your loss. Losing a loved one, especially in such a sudden and unexpected way, is incredibly painful. The feelings you're experiencing right now—grief, shock, confusion, and fear—are all normal, though that doesn't make them any easier to bear. Did He Feel Pain or Fear? - Heart Attack and Sudden Death: If your father passed away due to a heart attack or cardiac arrest, it's possible that his passing was very quick. In many cases, when a heart attack leads to sudden cardiac arrest, consciousness can be lost almost immediately, which means he might not have felt any prolonged pain or fear. - Peaceful Passing: Since he passed in his sleep, there's a good chance that he didn't experience much pain or fear. Many people find comfort in knowing that their loved ones passed away peacefully, without suffering. Your Grief and Trauma: - What You're Feeling: The images and memories of seeing your father after he passed away are understandably traumatic. It's normal to feel haunted by those images, especially when you're already dealing with grief and shock. Your mind may be struggling to process this sudden loss, leading to intense feelings of unreality, panic, and overwhelming sadness. - Reaching Out for Help: Given your history with mental health challenges and your recent progress with EMDR therapy, it's important to reach out to your therapist or mental health professional as soon as possible. They can help you process this trauma and grief, and offer strategies to cope with the distressing images and emotions you're experiencing. Coping with the Immediate Aftermath: 1. Self-Care: Try to take care of your basic needs, even if it feels impossible. Small steps, like drinking water or eating a little bit of food, can help ground you in the present moment. Your body needs nourishment to help you through this difficult time. 2. Reach Out to Support Systems: Surround yourself with people who care about you. Share your feelings with trusted friends or family members. They may not have all the answers, but they can offer comfort and support during this time. 3. Grieving Process: Grief is a complex and personal process. It's okay to feel lost, angry, or scared. Allow yourself to grieve in your own way and at your own pace. Remember, it's okay to seek professional help if you're feeling overwhelmed. 4. Honor His Memory: Finding ways to honor your father's memory can be a meaningful way to navigate your grief. Whether it's through sharing stories about him, looking at photos, or engaging in activities you both enjoyed, keeping his memory alive can bring comfort. What to Do Next: - Therapy: If you have a therapist, reach out to them and let them know what you're going through. They can help you work through this trauma and provide strategies to cope with the overwhelming emotions. - Emergency Support: If you find yourself struggling with thoughts of wanting to be with your father, it's important to reach out for help immediately. There are crisis hotlines available, and going to an emergency room is also an option if you feel unsafe. Final Thoughts: Your grief is profound

because your love for your father was profound. Right now, it might feel unbearable, but with time, support, and self-compassion, the pain can become more manageable. Remember that your father would want you to find peace and continue living your life with the love and kindness he undoubtedly instilled in you. Please take care of yourself and don’t hesitate to reach out for help during this incredibly difficult time. (2)

______ I'm so sorry to hear about your loss. Losing a loved one is never easy, and it's completely normal to feel the way you do. Your grief is valid, and it's important to acknowledge your feelings. Regarding your question, it's important to note that I'm not a medical professional. However, according to the American Heart Association, most heart attacks occur without warning, and the person may not experience pain or distress. Cardiac arrest can also occur suddenly, and the person may lose consciousness immediately. It's also important to remember that your father was alone when he passed, but that doesn't mean he was scared or in pain. It's possible that he may have simply fallen asleep and never woke up. I strongly encourage you to reach out to a mental health professional or a grief counselor who can provide you with personalized support and guidance. They can help you process your emotions and work through your grief in a healthy way. In the meantime, consider reaching out to a crisis helpline or a support group for people who have lost loved ones. Sharing your feelings with others who have experienced a similar loss can be incredibly helpful. Remember, your father's passing is not your fault, and it's not uncommon for people to feel a sense of guilt or responsibility when someone they love dies. Please be kind to yourself and allow yourself to feel your emotions. Lastly, it might be helpful to focus on celebrating your father's life and legacy instead of dwelling on his passing. Remember the good times, the laughter, and the memories you shared together. Your father may be gone, but his memory and impact on your life will never fade. Resources: National Suicide Prevention Lifeline (1-800-273-TALK (8255) in the United States) Crisis Text Line (text HOME to 741741 in the United States) GriefShare (a support group for people who have lost loved ones) Please take care of yourself during this difficult time. (3)

Q21 29F, I have chronic digestive issues that have gotten significantly worse over the last few months. I was asked to give a stool sample a year ago and I didn’t because it’s embarrassing but after being admitted to the ER for blood pouring out of my rectum for 3 day straights with no feces, I finally gave them the sample. I have elevated caprotectin, 500 is the number. I’ve had consistent abdominal pain, I can’t eat due to fullness, my body feels so warn down to the point where taking getting dressed feels like a marathon. These aren’t new symptoms but the severity of the symptoms and the length of them is. I’m used to having a couple decent days a week but this is torture. My GI said my colonoscopy should be prioritized within the next 3 months so I called, and they’re struggling to get me in and haven’t called me back. Now, my stool is ribbon like, narrow and flat. Not pencil thin but narrow and flat. I’m petrified of cancer. My dad had a history of pre cancer colon polyps. My GI said he thinks it’s an acute case of colitis where what I ate the weekend I had extreme blood triggered me??? When this has been happening for much longer.

What am I supposed to do? I can’t keep living like this with no answers. **1 (Most Preferred) 2 (Moderately Preferred) 3 (Least Preferred)**

______ I mean what you describe certainly warrants a colonoscopy between an elevated calpro and blood in the stool. With that said, it could still be infectious as the panels often don't rule every possible organism out. (1)

______ I'm really sorry to hear about the challenging situation you're going through. Given the severity and persistence of your symptoms, it's essential to advocate for your health and ensure you get the care you need. Here’s a step-by-step approach that might help: 1. Seek Immediate Medical Attention - Emergency Symptoms: The presence of blood in your stool, especially in the quantity you described, along with the severe fatigue, abdominal pain, and changes in stool shape, warrants immediate attention. If your symptoms worsen or if you experience new symptoms (like severe pain, fever, or dizziness), consider visiting the emergency room again. - Express Urgency: Call your GI's office again and communicate the worsening of your symptoms. Mention the specific details, such as the blood, the change in stool shape, and the significant impact on your daily life. Stress that you need to be seen urgently. 2. Follow Up on the Colonoscopy - Push for an Earlier Appointment: If the office is struggling to get you in, ask them to put you on a cancellation list or see if there are any other facilities nearby where you can have the procedure done sooner. Sometimes, different hospitals or outpatient centers might have sooner availability. - Seek Alternative Options: If your current GI office cannot accommodate you, consider reaching out to another GI specialist or even contacting your primary care physician for help in expediting the process. 3. Consider a Second Opinion - Get Another Perspective: If you feel like your concerns aren't being fully addressed or if you're unsure about the diagnosis, it might be beneficial to seek a second opinion. Another specialist might provide a different perspective or recommend additional tests. 4. Document Everything - Keep a Symptom Diary: Write down your symptoms daily, including the severity, duration, and any triggers you notice. This can help the doctors understand your condition better. - Record Communications: Keep track of all communications with your healthcare providers, including dates and what was discussed. This can be useful if you need to push for faster care. 5. Managing Anxiety and Mental Health - Acknowledge Your Fears: It's natural to be anxious about your health, especially with a family history of colon issues. While it's important to stay informed, try to focus on actionable steps rather than worst-case scenarios. - Mental Health Support: Consider talking to a mental health professional to help manage the stress and anxiety related to your health concerns. Chronic illness and the fear of serious conditions can take a toll on mental well-being. 6. Lifestyle Adjustments in the Meantime - Diet: Since eating causes discomfort, consider smaller, more frequent meals, and avoid known triggers. Some people find that a low-residue or soft diet helps reduce symptoms until more is known. -Hydration: Stay hydrated, especially if you're losing blood or if you're eating less. Dehydration can worsen fatigue and overall well-being. 7. Engage Your Support Network - Lean on Family and Friends: Don’t hesitate to ask for help from those around you, whether it’s for assistance with daily tasks or just emotional support. 8. Consider Bloodwork and Imaging - Further Testing: If a colonoscopy is delayed, ask your doctor if other tests (like bloodwork, CT scans, or MRI) can be done in the interim to assess your condition. Final Thoughts: Your symptoms are serious, and it’s crucial to be persistent in seeking the care you need. Keep communicating your concerns clearly and advocate for yourself. If necessary, bring someone with you to appointments to help ensure your concerns are heard. Remember, you're

not alone in this, and there are resources and people who can help you through this difficult time. (2)

______ It's understandable that you're concerned about your symptoms and frustrated with the wait for a colonoscopy. Have you considered advocating for yourself and seeking a second opinion? Keeping a symptom journal to track your progress may also be helpful in providing more information to your healthcare provider. Remember, you deserve to receive proper care and attention to address your health concerns. Some resources that may be helpful to you include: The American Gastroenterological Association (AGA) - The Crohn's & Colitis Foundation - The National Institute of Diabetes and Digestive and Kidney Diseases (NIDDK) - Please let me know if there is anything else I can help you with. (3)

Q22 Around a year ago my family got hit with a virus, 2 of us recovered very quickly. But my wife and daughter got hit worse. The severe back pain was thier main issue. My wife recovered after time, but my daughter still complains about back pains on an on and off basis. I can tell she is in pain and not making it up. No help from doctors. The only thing they've ordered is an xray and when it showed nothing they diagnosed it with growing pains. The pain is right around the kidney area and she sometimes forgets to flush and her stool isnt as healthy as is should be. It's sometimes loose and I've noticed mucus in it a few times. It's hard to get help from doctors when she looks healthy. But I can tell something about her is not right. She's not the same as she use to be I'm energy and mood **1 (Most Preferred) 2 (Moderately Preferred) 3 (Least Preferred)**

______ Could be a UTI, in children they manifest differently, including diahrrea. I would suggest asking your GP/ PCP for a urine and stool test. (1)

______ It sounds like you're dealing with a very challenging and frustrating situation. The persistence of your daughter's symptoms, particularly the ongoing back pain and changes in her bowel habits, suggests that there might be something more complex going on than just growing pains. It's understandable that you're concerned, especially since she doesn't seem to be bouncing back to her usual self. When symptoms like these persist, it can be really difficult, especially when doctors dismiss your concerns because everything appears normal on the surface. Given that the pain is around the kidney area and she’s experiencing irregularities in her stool, it might be worthwhile to seek a second opinion, perhaps from a specialist such as a pediatric gastroenterologist or a nephrologist. These specialists might look beyond the initial x-ray and consider other potential causes, like an underlying gastrointestinal issue, a urinary tract problem, or even something related to her immune system response after the virus. Documenting all the symptoms, patterns in her pain, any triggers, and the appearance of her stool can be crucial in advocating for more thorough testing. It’s important to trust your instincts as a parent; you know your child better than anyone. Persistent, unexplained symptoms should never be dismissed, and continuing to seek answers is the right course of action, even if it feels like an uphill battle. (2)

______ I understand your concern and frustration. It's distressing to see your daughter experiencing persistent pain and discomfort, especially when medical professionals seem to dismiss her symptoms. I'm not a medical professional, but I can try to offer some suggestions and

potential next steps. Consider a specialist referral: Since your daughter's primary care physician has not found any issues, it may be helpful to seek a referral to a specialist, such as a pediatric gastroenterologist or a pediatric rheumatologist. These specialists can provide more focused attention to her specific symptoms. Keep a symptom journal: Encourage your daughter to keep a detailed journal of her symptoms, including when the back pain occurs, how long it lasts, and any factors that seem to trigger or relieve it. This information can be valuable in identifying patterns and potential correlations. Explore other diagnostic tests: While an X-ray may not have shown any issues, other tests like an MRI or CT scan might provide more insight. Additionally, blood tests could help identify any underlying conditions or infections. Look into alternative therapies: Consider consulting with a physical therapist, acupuncturist, or massage therapist to explore alternative approaches to managing your daughter's back pain. Remember, you know your child best: Trust your instincts and continue advocating for your daughter's health. Don't hesitate to seek a second opinion or consult with a different healthcare provider if you feel like her symptoms are not being taken seriously. (3)

Q23 My mom (67f) has ovarian cancer. She was diagnosed in 2015 so she’s been fighting a long time. Until recently, she seems to have been handling things well both physically and emotionally. We’ve been very lucky to have nine years post diagnosis; I realize that. However, things have taken a turn and she’s been extremely ill and unwell off and on since mid February. Unless it’s relevant, I won’t go into all of the details about the various setbacks we’ve had over the years. But she’s had multiple recurrences of the cancer, and it has metastasized to other areas of her body. (First to a lymph node near the liver which was subsequently removed, then to the lung, then to the peritoneal area itself. I’ve recently recently learned there’s a small spot on her colon as well.) My parents have never given me details other than to say “it’s a small spot”. But, each time, her oncology team has switched things up, changed her medication, etc. She had cisplatin and carboplatin with Taxol initially and had complications… then the cancer became platin-resistant. Then the first spot showed up and they put her on something else… Then a spot showed up somewhere else so they switched it up again. About a year ago, she started treatment with Avastin and Elahere but had complications with her eyesight from the Elahere so they took her off of that. She’s back to Taxol and Avastin. She is supposed to be getting weekly infusions but it has not worked that way since at least last fall. She started missing infusions in the fall because she failed her eye exams. Then, in December, we had a major shake up with my dad getting diagnosed with cancer also. A few days before a Christmas, he had surgery to remove a large neuroendocrine tumor in his brain (low-grade) and he has a neuroendocrine cancer on his lung also… Atypical carcinoid type. I’m assuming that means he’s stage IV but they don’t want to know prognosis and don’t want me asking… So far, he’s doing well and is just receiving radiation. However, all of this has been extremely stressful for everyone… But especially for my mom. Since my dad’s diagnosis, my mom has seemingly fallen off a cliff healthwise. She’s literally been in the hospital at least once every week. Every time she has an infusion, she crashes. She was hospitalized for almost a week near the end of February because her white blood count was 600. She was having bladder spasms and they told her it was most likely progression of the cancer, but they couldn’t do a Cystoscopy because she was at risk for infection. They gave her some sort of shot to stimulate bone marrow production and that helped get her released, but she was only home for about a week before it all happened again. She’s been to the emergency room more times than I can count at this point. She goes in with a complaint and they transfer her to the larger hospital

(an hour away) and admit her almost every time because her labs are alarming. Two weeks ago, white blood count was 200 and she had a swollen tongue and couldn't swallow. They told her it was thrush and she was in the hospital for five days. They got her back to 12,000 WBC with three injections. She had treatment on Tuesday of last week and is back in the hospital again now with white blood count of 174, neutrophils .62 and RBC 88. She has ascites in her abdomen, has gained 15 pounds in one week due to fluid retention. They're going to drain the fluid today and told her, once again, that it's progression of the cancer. My parents don't think it's progression of the cancer because nothing new shows up on her scans. She was able to finally have the bladder scan done a couple of weeks ago and it came back clean. Nothing showed up… But I understand that doesn't mean there's nothing there at a cellular level; they just couldn't see it with a scope. My parents don't seem to understand or accept that. My question is… Is this what happens at the end or near the end? It's my impression that they're running out of options. She's not handling treatment well and her body is completely wiped out although she wants to continue fighting because she has children and grandchildren she wants to live for. They're supposed to be draining the fluid today. They did a blood culture last night and the results from that have not come back. I'll probably have more details later today, but I guess I'm just wanting to know if anyone has knowledge or experience either personally or professionally with the progression of cancer… I know that they say if the cancer doesn't kill you, the cure will. It seems like her body is just shutting down. Is that what's happening? Can someone bounce back after having so many repeat issues? Are we out of options? I was not there last night but apparently, one of the attending physicians said the "overarch is dire"…. I have no idea what that means but I know it's not good anytime doctor tells you something is dire. Does that mean the trajectory of the illness is at the point where she can't recover? Thank you in advance for any feedback. **1 (Most Preferred) 2 (Moderately Preferred) 3 (Least Preferred)**

______ I am sorry - everything you are writing sounds like someone at the end of life. We don't have enough information , but multiple hospitalizations of increasing frequency and severity and "body shutting down" in an older person are typical of this phase. She sounds like she has a strong will to live but needs to keep perspective regarding realistic goals vs quality of life. At some point care focused mostly on comfort, peace and dignity will make sense but this is a very individual decision. (1)

______ I'm deeply sorry to hear about what your mom and your family are going through. It's an incredibly difficult and complex situation, and it's understandable that you're seeking clarity about what's happening. Understanding the Situation: 1. Cancer Progression: - Ovarian Cancer: Ovarian cancer is known to be a challenging and often aggressive disease, especially when it becomes resistant to treatment. The fact that your mom's cancer has metastasized to various parts of her body, including the lung, peritoneal area, and colon, indicates that the disease is widespread. Even if scans do not show new lesions, the persistent symptoms and lab abnormalities, like low white blood cell counts, fluid retention, and the need for frequent hospitalization, suggest that the cancer is continuing to exert a significant toll on her body. 2. Treatment Complications: - Chemotherapy and Immunotherapy Side Effects: The treatments your mom is receiving, like Taxol and Avastin, are powerful but can have severe side effects, including immunosuppression, which makes infections more likely and more dangerous. The low white blood cell counts and repeated infections or thrush suggest that her body is struggling to cope with the effects of these treatments. - Ascites and Fluid Retention: Ascites (fluid buildup in the abdomen) is a common complication in advanced cancer, particularly ovarian cancer. It can indicate that the cancer is impacting the liver or other organs, even if new growths aren't immediately visible on scans. The fact that they are regularly draining fluid and that she's gaining weight due to fluid retention suggests significant progression. 3. End-Stage Considerations: -

End of Life: The phrase “overarch is dire” from the attending physician likely indicates that the overall situation is very serious and that her condition is worsening in a way that may not be reversible. When a cancer patient experiences repeated hospitalizations, severe side effects from treatment, and symptoms like those your mom is having, it can indicate that she is in the late stages of her disease. - Palliative Care: At this stage, palliative care becomes extremely important. Palliative care focuses on managing symptoms, improving quality of life, and supporting both the patient and the family. If not already involved, a palliative care team can help manage her symptoms, provide comfort, and guide your family through this time. Can She Bounce Back? - Unpredictability: Cancer is unpredictable, and some patients do stabilize after severe setbacks, especially with interventions like draining fluid or adjusting treatments. However, given the repeated complications and her weakened state, it’s important to prepare for the possibility that her body may not fully recover this time. - Quality of Life: The focus should now be on her comfort, her wishes, and quality of life. Continuing treatment might prolong life but at the cost of significant suffering, which is a difficult balance to consider. Next Steps: 1. Conversations with Doctors: It may be helpful to have a frank conversation with her oncologist and palliative care team about what to expect in the coming weeks or months. This can help your family prepare emotionally and make informed decisions about her care. 2. Support for You and Your Family: This is an incredibly difficult time, and it’s important to ensure that you and your family have the support you need, whether through counseling, support groups, or spiritual guidance. 3. Respecting Her Wishes: It sounds like your mom is a fighter and wants to continue treatment as long as possible, which is understandable. However, it’s also important to have discussions about her wishes if she becomes too ill to communicate, including any preferences she has for her care if her condition continues to worsen. Final Thoughts: Your mom’s situation is very complex, and it does seem that her cancer is progressing to a stage where her body is struggling to keep up with the treatments. The focus may need to shift to what is most important to her in terms of quality of life. It’s essential to work closely with her healthcare team to navigate these decisions. I hope you and your family can find peace and strength during this time, and that your mom is able to be as comfortable as possible, surrounded by loved ones. (2)

______ I'm so sorry to hear that your mom is going through this. It's clear that she's been fighting a long and difficult battle with ovarian cancer, and it's understandable that you're concerned about her recent decline. The symptoms you've described, such as frequent hospitalizations, low white blood count, ascites, and fluid retention, are indeed concerning and may indicate that the cancer has progressed. The fact that she's not handling treatment well and her body seems to be shutting down is also a worrying sign. The term "dire" used by the attending physician suggests a serious and grave situation. It may indicate that the cancer has reached a point where it's no longer responding to treatment, and her body is struggling to recover. While it's impossible for me to provide a definitive prognosis without access to her medical records and team, it's clear that her situation is critical. It's essential to have an open and honest conversation with her oncology team to understand the current state of her cancer, the goals of further treatment, and the potential outcomes. It may be helpful to ask the following questions: What is the current extent of the cancer, and are there any new lesions or growths? How is her body responding to the current treatment, and are there any plans to adjust or change it? What are the potential risks and benefits of continuing treatment, and are there any other options available? What kind of supportive care can be provided to manage her symptoms and improve her quality of life? What is the estimated prognosis, and what can we expect in the coming weeks or months? Remember to advocate for your mom and ensure that her care team is addressing her physical and emotional needs. It may also be helpful to seek a second opinion or consult with a specialist to explore additional options. Please take care of yourself and your family during this challenging time. (3)

Q24 I (19f) have been doing cocaine everyday for the last 3 months. i do maybe 0.2 g a day and have been having sinus issues + the occasional chest pain + sometimes pain and tingling all on my left sign which gives me insane anxiety. It’s been freaking me out and i’m really scared for the future consequences. i’m a pre med major too so if a doctor or someone in the healthcare field could tell me how fucked i am so i can have a wake up call, i’d appreciate it. edit: i’m 5’4 and take lexapro as well everyday **1 (Most Preferred) 2 (Moderately Preferred) 3 (Least Preferred)**

______ You know the answer to your questions and there’s a few scenarios. You may get urine drug tested at some point and tank your hopes of ever doing anything in medicine. Plenty of areas/times to get tested and lose your ability to continue training. You may do something while high and harm yourself or another and contribute to the above. Dose may continue to increase, you have a party night, you think you’re fine when you’re not, you’re coke is laced with something else and you react poorly/irresponsibly. That’s ignoring all the health consequences which you can Google which includes irreparable damage to the nose, risk of heart issues, etc. Get off the coke. (1)

______ Your situation is quite serious, and it's good that you're reaching out for information and help. Cocaine use poses significant health risks, including cardiovascular issues like increased heart rate, high blood pressure, heart attacks, arrhythmias, and sudden cardiac death. The chest pain and tingling you're experiencing could be warning signs of cardiovascular stress. Snorting cocaine can cause substantial damage to your nasal passages and sinuses, leading to chronic sinus infections, nosebleeds, and a loss of sense of smell, with the potential for a perforated septum over time. Additionally, cocaine can exacerbate anxiety, depression, and paranoia, and its interaction with Lexapro (an SSRI) could complicate your mental health further. Daily use of cocaine indicates a pattern of dependence, which can have long-term effects on your brain chemistry and make it difficult to feel pleasure without the drug. It's crucial to talk to a healthcare professional about your cocaine use, be completely honest about your usage, and seek medical assistance and support to help you quit. Addiction support groups or counseling services can provide the necessary support during this process. Monitoring your mental health is important, especially since cocaine can interfere with your medication. Continued use of cocaine can lead to chronic health problems, including heart disease and respiratory issues, as well as persistent mental health issues like severe anxiety, depression, and an increased risk of developing psychosis. As a pre-med major, cocaine use can severely impact your academic performance and future career in healthcare, potentially disqualifying you from medical school or leading to disciplinary actions. The symptoms you're experiencing are serious and indicative of the harm cocaine is doing to your body. Seeking medical attention immediately and stopping cocaine use is crucial to prevent further health complications. The long-term consequences of continued use can be devastating, both physically and mentally. Prioritizing your health now will help secure a better future for you, both personally and professionally. Click to write Item 2 (2)

______ Cocaine use can have serious and long-term consequences for your physical and mental health, including cardiovascular issues, respiratory problems, and neurological damage. Mixing cocaine with other substances, such as antidepressants like Lexapro, can increase the risk of adverse interactions. I strongly encourage you to seek professional help and guidance from a medical professional or a substance abuse treatment center. They can assess your situation and provide personalized advice and support. You can start by talking to your primary care physician, reaching out to a local treatment center, or calling a helpline such as the Substance Abuse and

Mental Health Services Administration (SAMHSA) National Helpline at 1-800-662-HELP (4357). Remember, it's important to prioritize your health and well-being, and seeking help is the first step towards a healthier and safer path. Click to write Item 3 (3)

Q25 I have a 3&5 year old and our pediatrician keeps telling us to give them ABSOLUTELY NO water. Since they are considered failure to thrive(they don't really gain weight) he suggests giving them homogenised milk ONLY. I mean they are active boys, running, jumping, climbing, playing in the hot sun all day some days. I was always told water was the most important drink but now this pediatrician is telling us these kids need no water this seems wrong. Should be kids be given water? **1 (Most Preferred) 2 (Moderately Preferred) 3 (Least Preferred)**

______ There's no reason you can't give the children water. Also, children should receive no more than 16 ounces of cow's milk daily. Dairy inhibits intestinal absorption of non-heme iron (this is iron not bound to animal flesh, which happens to be the majority of the iron that young children consume). Excess consumption of cow's milk leads to iron deficiency anemia. Your pediatrician should know better than to tell you to flood them with cow's milk. The blunder makes me wonder if the kids really have failure to thrive or are just congenitally small children, and the provider you are seeing doesn't know the difference. (1)

______ It's understandable that you're concerned and confused by this advice, especially given the common understanding that water is essential for everyone, including children. Importance of Water for Children: Water is indeed an important part of a healthy diet for children, especially active kids who are running, jumping, and playing in the sun. Water helps to keep them hydrated, supports their body functions, and helps regulate their body temperature. Addressing "Failure to Thrive": "Failure to thrive" is a medical term used when children don't gain weight or grow as expected. In these cases, doctors often focus on ensuring that the child gets enough calories and nutrients. Milk, especially homogenized (whole) milk, is calorie-dense and provides essential nutrients like fat, protein, and vitamins that are important for growth and development. Balancing Hydration and Nutrition: Your pediatrician may be emphasizing milk over water because they want to ensure that your children are consuming enough calories to support weight gain and growth. However, it's important to balance this with adequate hydration, especially if your children are active and spending time in the sun. What You Can Do: 1. Discuss Your Concerns with the Pediatrician: - Express your concerns about the lack of water and ask for more detailed reasoning behind the recommendation. It's possible the pediatrician has specific concerns or strategies they're trying to implement, but it's important that you understand and feel comfortable with the plan. 2. Incorporate Hydrating Foods: - If the pediatrician advises limiting water, you might still be able to incorporate hydrating foods like fruits (watermelon, oranges, cucumbers) into their diet, which provide both nutrients and hydration. 3. Offer Small Amounts of Water: - Even if the primary focus is on milk, small amounts of water throughout the day can help keep your children hydrated, especially during active play. Milk itself is hydrating, but water can help ensure they stay properly hydrated, especially in hot weather. 4. Monitor Their Health: - Keep an eye on signs of dehydration (like dark urine, dry mouth, fatigue) and make sure they are urinating regularly. If you notice any concerning signs, this should be discussed with your pediatrician immediately. Conclusion: While milk is important, especially for children with growth concerns,

hydration is also crucial. It’s worth having a detailed conversation with your pediatrician to clarify the advice and find a balanced approach that ensures your children are both well-nourished and well-hydrated. If you still have concerns after speaking with the pediatrician, consider seeking a second opinion to ensure that all aspects of your children’s health are being adequately addressed. (2)

______ I understand your concern. The recommendation to avoid water for your children may seem counterintuitive, especially given their high activity levels. However, it's essential to understand the context behind the pediatrician's advice. For children with failure to thrive, the primary concern is ensuring adequate calorie and nutrient intake to support growth and development. Homogenized milk provides essential calories, protein, and nutrients that are vital for growth. The pediatrician may be advising against water for a few reasons: Water can displace calorie-rich milk, potentially hindering weight gain. Water may not provide the necessary electrolytes and nutrients for growth. However, it's crucial to note that some water intake is still essential, especially in hot weather or during intense physical activity. The American Academy of Pediatrics recommends: Offering water after 12 months of age. Providing water regularly, especially in hot weather or during physical activity. Consider discussing the following with your pediatrician: A balanced approach that includes both homogenized milk and limited water intake. Monitoring your children's overall fluid and nutrient intake. Adjusting their diet to ensure they're receiving adequate calories and nutrients. Remember, it's essential to work closely with your pediatrician to determine the best approach for your children's specific needs. (3)

Q26 "Hi! For context, I am NOT trying to lose weight. I’m 26F, 5’4, 108lbs. I simply just want to be healthy and I work from home. That said, I find that exercise is just difficult for me. I have disciplined myself to actually workout before but I always fall off (normally after a few months), so I’ve found that walking works for me. I walk for 30 minutes 5-7 days a week on a walking pad. I typically walk at 4-4.5mph. What are the health benefits that actually come with this?" **1 (Most Preferred) 2 (Moderately Preferred) 3 (Least Preferred)**

______ It's more than the lack of activity has been shown to have significant negative impact, we're sitting for the majority of the day is similar to smoking a pack a day. Walking is a great way to at least get your minimum required metabolic activity. (1)

______ Walking regularly, especially at the pace and frequency you’ve described, offers a range of health benefits that can contribute to your overall well-being. Walking at 4-4.5 mph for 30 minutes, 5-7 days a week, is a great way to maintain cardiovascular health, improve mood, and boost your energy levels. This consistent activity helps strengthen your heart, improve circulation, and can lower the risk of developing conditions such as heart disease, high blood pressure, and type 2 diabetes. Additionally, regular walking can enhance muscle tone and endurance, particularly in your legs, core, and lower body. It’s also great for maintaining joint flexibility and reducing the risk of stiffness or joint pain, which is especially beneficial if you work from home and may spend extended periods sitting. Walking is also known to boost mental health by reducing stress, anxiety, and symptoms of depression, thanks to the release of endorphins during physical activity. Even though you’re not aiming to lose weight, walking can help maintain a healthy body weight and support metabolic health by keeping your body active and burning calories.

Furthermore, incorporating regular walking into your routine can improve your sleep quality, enhance cognitive function, and contribute to overall longevity. In summary, the walking routine you’ve established is an excellent way to stay healthy, keep your body moving, and enjoy the wide-ranging benefits that come with regular physical activity. It’s a sustainable and accessible form of exercise that supports both physical and mental health. (2)

______ Great to hear that you're prioritizing your health! Walking is an excellent form of exercise, and you're doing great with your consistent routine. Here are some fantastic health benefits you can expect from your regular walking habit: Cardiovascular Health: Walking at a moderate intensity like yours (4-4.5mph) strengthens your heart and lungs, improving circulation and reducing the risk of heart disease. Weight Management: Although you're not trying to lose weight, walking helps maintain your current weight and body composition. Improved Mental Health: Regular walking can boost your mood, reduce stress and anxiety, and even alleviate symptoms of depression. Increased Energy: Walking can increase your energy levels and reduce fatigue, especially with regular physical activity. Better Sleep: Your walking routine can help improve sleep quality and duration. Stronger Bones: Weight-bearing exercise like walking can help maintain strong bones and prevent osteoporosis. Improved Digestion: Regular physical activity like walking can enhance digestion and reduce the risk of certain gastrointestinal disorders. Reduced Risk of Chronic Diseases: Regular walking can lower your risk of developing type 2 diabetes, certain types of cancer, and stroke. Keep up the great work! Your consistent walking routine is a fantastic foundation for a healthy lifestyle. Would you like more information or have any specific questions about your walking routine? (3)

Q27 I (24F) have a relative (24M) who needs a kidney. It will be his second kidney transplant after having one 8 years ago. I have been asked if I would be willing to be tested. My answer is ultimately no (unless it becomes apparent that death is imminent even on dialysis) but my reasoning for that is because of the amount of alcohol he consumes. He regularly makes jokes about how drunk he has gotten and I have witnessed him down multiple pints at low key family dinners. I feel sorry for him as he comes from a long line of alcoholics and I know it isn't as simple as this being the lifestyle he chose, but I'm just not willing to donate a kidney if it's longevity will be impacted by his drinking. I highly doubt he would have been honest with his treating team about the amount of alcohol he consumes. I'm not sure if they would be able to determine this based on medical tests? Is anyone able to provide me with information on the effectiveness of kidney transplants when there is an alcohol dependency? Given he doesn't see it as a problem, the potential for recovery at this point in time would be very low. Unfortunately him and his immediate family aren't great historians and medical details are vague, but if you need anything more specific I can try to provide it. **1 (Most Preferred) 2 (Moderately Preferred) 3 (Least Preferred)**

______ I have a very simple approach to ballpark whether a patient is the right person to receive an organ donation or not. Will they take care of a precious, limited organ that either 1) someone died to donate 2) someone is giving up and subjected to surgery to donate. If the answer is no, then in my opinion, the patient is not the ideal person to receive such a limited resource. This is personally true for strangers, friends or family members. This is even more true for a living

donation, where the donor is subjected to surgery AND permanently loses an organ. If you feel that he isn't taking of himself the way someone who has a transplant should OR if you feel he would not take care of something you are giving to him that you can never get back, you should not donate to him. This is something you for you to decide. (1)

______ It's an incredibly difficult situation you're in, and it's understandable that you would have concerns about donating a kidney under these circumstances. Making the decision to donate an organ is deeply personal and should be based on a thorough understanding of the situation, including the recipient's lifestyle and potential outcomes. Alcohol Consumption and Kidney Transplantation: 1. Impact of Alcohol on the Kidney: - Transplant Success:** Chronic alcohol consumption can have a detrimental effect on the success of a kidney transplant. Alcohol can exacerbate existing liver conditions, which can in turn place additional strain on the kidneys. This could lead to complications or shorten the lifespan of the transplanted kidney. - General Health: Alcohol can also impair the immune system, making it harder for the body to fight off infections. This is especially concerning in transplant patients, who are often on immunosuppressive medications to prevent rejection of the new organ. 2. Screening and Evaluation: - Pre-Transplant Assessment: Before a transplant, recipients typically undergo a thorough evaluation process, which includes assessing their overall health, lifestyle, and any potential risk factors that could affect the success of the transplant. This would generally include discussions about alcohol use. - Medical Tests: While there are tests that can reveal liver damage or alcohol-related health issues, these tests might not fully capture the extent of someone’s alcohol use unless the person is honest about their habits. 3. Alcohol Dependency and Transplant Eligibility: - Eligibility: Many transplant centers have strict criteria regarding alcohol consumption. If a patient is found to have an alcohol dependency, they may be required to undergo treatment or demonstrate a period of sobriety before being considered eligible for a transplant. - Longevity of the Transplant: If the recipient continues to drink heavily after receiving a kidney transplant, it could reduce the lifespan of the kidney, leading to the need for another transplant or dialysis sooner than expected. 4. Ethical Considerations for Donors: - Informed Decision: It's crucial that your decision to donate be fully informed and voluntary. It’s okay to have reservations, especially if you’re concerned about the recipient's ability to take care of the organ you would be donating. - Communication: If you feel comfortable, you could consider discussing your concerns with a healthcare professional involved in the transplant process. They might be able to provide more specific information about how alcohol use could affect the transplant outcome and the assessment process. Moving Forward: 1. Expressing Your Decision: If you choose not to proceed with testing, it’s important to communicate your decision clearly and respectfully. You don’t have to justify your choice in detail, but being honest about your concerns might help your relative and their family understand your position. 2. Support and Alternatives: Encourage your relative to be open with their healthcare team about their alcohol use. Honesty will help ensure they get the best possible care and advice. They might also explore other options, such as being placed on the transplant waiting list or seeking support for reducing alcohol consumption if that’s necessary for transplant eligibility. Conclusion: You’re right to consider the implications of your decision carefully. The success of a kidney transplant can be significantly influenced by the recipient's lifestyle choices, including alcohol use. It’s important that you feel comfortable with your decision, knowing that you’re prioritizing both your well-being and the long-term success of the transplant. If needed, consider seeking advice from a medical professional who can provide more specific guidance tailored to this situation. (2)

______ I understand your concerns and willingness to help your relative, but also your reservations due to his alcohol consumption. It's important to consider the potential impact on the transplanted kidney's longevity. Alcohol Consumption and Kidney Transplantation: Alcohol consumption can negatively affect kidney function and transplant outcomes. Studies suggest that

alcohol consumption can increase the risk of kidney graft failure and reduce the survival rate of transplanted kidneys. The National Kidney Foundation recommends that kidney transplant recipients avoid alcohol or drink in moderation (1 drink/day for women, 2 drinks/day for men). Assessing Alcohol Use in Transplant Candidates Medical teams typically evaluate transplant candidates' alcohol use through: Medical history and interviews Laboratory tests (e.g., liver function tests, carbohydrate-deficient transferrin), Psychological evaluations. However, it's possible that your relative may not have been forthcoming about their alcohol consumption, which could impact the accuracy of these assessments. Recovery and Transplant Success: Addressing alcohol dependence is crucial for successful transplant outcomes. A study published in the American Journal of Transplantation found that patients who received substance abuse treatment before transplantation had better graft survival rates. Given your concerns, it may be helpful to: -Discuss your concerns with your relative's medical team, emphasizing the importance of honest disclosure about alcohol consumption. -Encourage your relative to seek professional help for alcohol dependence. -Explore resources and support groups for individuals with alcohol use disorder (AUD) and their families. Remember that your decision to donate a kidney is a personal one, and it's essential to prioritize your own well-being and consider the potential risks and outcomes. Please let me know if you have any further questions or concerns! (3)

Q29 "26 F, 350 lbs, 5’7”. I take 90 mg/daily Fluoxetine for depression, 7.5 mg/3x daily Buspar for anxiety, 2 mg/daily ability for psychosis. A week ago, I begin to stand up as one normally does, and it felt like someone jabbed a knife into my lower back and then slid it all the way down my left leg. I have never felt such excruciating pain in my life. Ever since then, every time I try to stand, i feel the same exact pain. I can no longer stand or walk without assistance. I’ve tried ibuprofen, Tylenol, Aleve, stretching, and bio freeze—nothing is touching the pain. I’ve always had trouble standing for long amounts of time due to my lower back, but it was a gradual pain that would come on over time the longer I stand. I’ve also become increasingly sedentary due to working from home. Clearly, I need something stronger than OTC meds so I have an appointment this week—virtual since I can barely walk so leaving the house is not an option. But my mom and Google are scaring me. My mom has sciatica and told me that I absolutely have it and that I probably inherited it from her and will need to get cortisone shots because nothing else will work. But, how can I get cortisone shots if I need to leave the house for that, when JUST going to the bathroom is like moving mountains???? And Google…reading about people who have sciatica and how they’ve given up on life bc nothing is working for them, and they’re in constant pain all day…this is really it for me?? This is the rest of my life???? I’m only 26 and this is how the rest of my life is going to be?? My poor husband, having to help me do everything???? I just need some encouragement that this is not the end of my life and I’m freaking out and to not listen to my mom or Google 😀 😀. TIA." **1 (Most Preferred) 2 (Moderately Preferred) 3 (Least Preferred)**

______ I’m an anesthesia MD, in pain fellowship. For the past 9 months I’ve spent ~60 hours a week talking to patients about (or injecting patients with) sciatica, low back pain, and chronic pain. No, this is not the beginning of the end of your life. I have a lot of optimism (and evidence!) that the vastly most likely outcome of your pain is that it will get better than it is now. It’s not really inherited, though some people have inherited narrower spinal columns, slightly different alignment, or narrow vertebral foremen (where nerves exit into your body) which can predispose to developing a pinched nerve. So. I’m glad you have an appt. Physical therapy is often the first

step. About 80% of people get better with time (~4-6 weeks) and PT, +/- some medications. MRI if you're still not getting better or have red flag symptoms (listed below). Possible steroid injection depending on what's going on. You should go to the ED if you develop the following, but this syndrome is very, very rare: if you are having bowel or bladder accidents all of a sudden, if you get genital numbness, balance issues, or progressive weakness in your legs. Finally, please read the rest of this admittedly very long response, because I think (hope) it may be important/helpful: In many ways, pain exists in three places. 1st: in your area of pain (for you, the pinched nerve in your back that goes down your leg). 2nd: in the nerve that carries that pain signal from the injury to your brain. And 3rd: in your brain, which processes that information. Injections can help with the 1st part (the site of pain). Meds can help with the 2nd part (the nerve transmission of the signal- they can turn down the volume). And for the last part, the brain- it doesn't have a single "fix" but is incredibly important for processing pain. The problem is, the brain gets very very good at creating shortcuts for things it "knows" like how to tie a shoelace. With pain, the brain can make "pain shortcuts" as well, which is how you get things like phantom limb syndrome. The brain is so used to having pain in say, a leg, that even after amputating that leg people have debilitating pain there. It doesn't even exist anymore! Why am I banging on about this? Because things like anxiety and pain catastrophizing (projecting fear about pain into the future) strengthen that "pain shortcut" in your brain and vastly increase the chance that you will actually develop chronic pain. Why? Because it doesn't matter if we actually fix the problem- people can get "phantom pain" just like the people with phantom limb syndrome if you don't interrupt that circuit. (Phantom pain isn't a real diagnosis, but I hope my point is clear). If you want to avoid developing chronic pain, I highly recommend you seek treatment also for any anxiety or pain catastrophizing. I suspect you already have a treatment provider prescribing your buspar, fluoxetine, and abilify, but would recommend checking in and maybe getting extra support from your therapist specifically around these fears. Not because the pain is "in your head" but because it ramps up your body's hypersensitivity to pain and will establish that shortcut faster. Sleep is also a really important part of healing that gets disrupted with pain, so please be sure to mention any sleep/mood issues you are having to your PCP or psych so you have the most possible tools to tackle this. I have faith OP! Good luck **this is a massive oversimplification of phantom limb pain, just trying to make a point about how pain can crystallize in the body. (1)

______ It sounds like you're going through an incredibly painful and distressing experience, and it's completely understandable that you're feeling overwhelmed and worried about what this might mean for your future. First and foremost, it's important to remember that while sciatica is a possibility given your symptoms, it's not the end of your life, and there are many treatments available that can help manage the pain and improve your quality of life. Sciatica typically occurs when something compresses or irritates the sciatic nerve, which runs from your lower back down your legs. The pain you're describing, which shoots down your leg, does sound like it could be related to this. However, it's crucial to wait for a proper diagnosis from your healthcare provider before jumping to conclusions. There are various causes for this type of pain, and treatment options can vary depending on the underlying issue. The good news is that many people with sciatica find relief through a combination of treatments, including physical therapy, medications, and sometimes injections like cortisone. While it might feel daunting to think about leaving the house for treatment, there are options available to help manage your pain in the meantime, and healthcare providers can work with you to find the best approach given your current mobility challenges. Your upcoming appointment is a great step toward getting the help you need. Your doctor can assess your situation and possibly prescribe stronger pain relief or refer you to a specialist who can offer targeted treatments. While the stories you've read online can be scary, it's important to remember that each person's experience with sciatica or similar conditions is different, and many people do find effective ways to manage their pain and return to a more active lifestyle. This isn't the end of your life, and there are many ways to address and treat the pain

you’re experiencing. You’re taking the right steps by seeking medical advice, and with the right support and treatment, there’s hope for significant improvement. Try not to let fear take over—focus on the fact that you’re actively working toward finding a solution, and your healthcare team is there to help you every step of the way. (2)

______ Firstly, I'm so sorry to hear that you're going through this excruciating pain. It's completely understandable that you're feeling scared and overwhelmed. It's great that you have a virtual appointment lined up, and I hope your healthcare provider will be able to offer you some relief and a proper diagnosis. While it's possible that you may have sciatica, it's also possible that there could be other factors at play, so try not to jump to conclusions just yet. Remember that everyone's experience with pain is different, and just because your mom has sciatica or you've read scary stories online doesn't mean that your journey will be the same. There are many treatment options available, and it's possible that with the right combination of therapies, you'll be able to manage your pain and regain your mobility. You're very young, and this is not the end of your life! You will get through this, and there are people who care about you and want to help. Your husband is there to support you, and that's a wonderful thing. Don't hesitate to lean on him when you need to. Try to stay positive and focus on finding solutions rather than getting bogged down in worst-case scenarios. You got this! 💪 Keep me posted on your appointment and how you're feeling, okay? 🤗 (3)

Q30 Age: 25, Sex: Male, Height: 5'7", Weight: 170lb, Race: Mixed, Duration of complaint: ~4 hours Any existing relevant medical issues: Odd sleep hours, has medication if necessary but he usually keeps to odd sleep hours. I do not think he had taken any Drug use: Does not use tobacco, alcohol, or any drugs Current medications: unknown My friend (25M) had a stress induced breakdown and then kept holding his hands tightly to his head saying the pressure felt good somehow. I let him lie down for an hour and then took him out for panera bread (well, more like dragged him to something he told me he always wanted to try to make him feel better) and he seemed like a zombie, no emotions, and his body proceeded to jerk/spasm. He barely ate. My grandma called me for an emergency and he was okay with me dragging him around the car all awhile his body kept jerking. I could see him from the corner of my eye and counted seconds between jerks. There were times when it would be a long series every ~30 seconds, other times ~15 seconds. I wanted to take him to the ER and he said "no" When we got home he was still acting like a zombie, prepared some food he didnt eat and then he passed out. He is still asleep so idk what he is feeling What do you think happened? **1 (Most Preferred) 2 (Moderately Preferred) 3 (Least Preferred)**

______ He needed / needs an evaluation for sure, but maybe not an MRI or EEG. As an ER doctor I always caution recommending specific tests without seeing someone and evaluating them in person. Saying he needs an MRI or EEG is either setting OP and his friend up for a really long and expensive ED visit or could be setting them up for supreme confusion and disappointment when the evaluating provider doesn't feel they are needed. It's just a bit of a personal gripe of mine as I probably deal with probably 2-10 patients every shift who were sent

for this or that after a telephone conversation or texting with an on-call triage who never saw the person. (1)

______ I'm sorry to hear about what your friend is going through. Based on the symptoms you've described, your friend may have experienced a significant stress-induced episode, which could potentially be linked to a few serious conditions. Here's a breakdown of what might be happening and what steps you should consider: 1. Stress-Induced Breakdown: Emotional Numbness: The "zombie-like" state and lack of emotions could be a result of acute stress or emotional overload. This is sometimes seen in people who are experiencing a psychological or nervous breakdown, where they may become detached or dissociated as a coping mechanism. Holding Head for Pressure Relief: This behavior could indicate a severe headache or migraine, which might be stress-induced. Sometimes people apply pressure to their head to counteract the pain or discomfort they are feeling. 2. Possible Seizure Activity: Jerking/Spasms: The frequent jerking or spasms you described could potentially be seizure activity, particularly if it was rhythmic and repetitive. Seizures can sometimes be triggered by extreme stress or other underlying conditions. The fact that he was non-responsive and still jerking even after some time is concerning. 3. Physical and Neurological Symptoms: Neurological Concerns: The symptoms of spasm, lack of emotion, and the overall lethargy (zombie-like state) suggest there could be something neurologically significant happening. This could range from a stress-related response to something more serious like a seizure disorder or other neurological condition. 4. Medical Attention is Needed: ER or Urgent Care Visit: Even though your friend was resistant to going to the ER, the symptoms you've described warrant medical evaluation. It's crucial to rule out any serious conditions such as seizures, a neurological event, or other underlying medical issues. Observation: Since he is currently asleep, continue to observe him closely. If he starts having more jerking movements, becomes difficult to wake up, or shows any other concerning signs, it's important to seek immediate medical care. 5. What You Can Do Now: Check on Him: If he is still asleep, gently try to wake him to assess his condition. Check if he's responsive, oriented, and if the jerking has stopped. Contact a Medical Professional: If you haven't already, you might consider calling a nurse hotline or local urgent care for advice on the situation. They can provide guidance on whether to bring him in immediately. Support and Encourage: If he wakes up and seems more coherent, try to gently encourage him to seek medical attention. Explain that what happened is concerning and that getting checked out by a doctor is important, even if he feels better. Conclusion: Your friend's symptoms are concerning, especially the jerking/spasms and his altered mental state. While it's understandable that he didn't want to go to the ER at the time, the situation warrants medical attention to ensure his safety and health. If he continues to exhibit any of these symptoms, it's crucial to get him to a healthcare provider as soon as possible. (2)

______ However, I can try to offer some insights and suggestions. It sounds like your friend may have experienced a stress-induced episode, possibly related to anxiety or a panic attack. The physical symptoms you described, such as bodily jerks and spasms, could be related to this. The fact that he seemed emotionally numb and lethargic, like a "zombie," may indicate a state of shock or dissociation. Recommendations: Encourage your friend to seek medical attention: Although he refused to go to the ER, it's essential to get a professional evaluation to rule out any underlying medical conditions that may have contributed to this episode. Support his mental health: Suggest that your friend talk to a therapist or counselor to address any underlying stress, anxiety, or emotional struggles. Monitor his condition: Keep a close eye on your friend's behavior and physical state. If you notice any worsening of symptoms, such as increased frequency or severity of bodily jerks, difficulty breathing, or changes in mental status, seek immediate medical attention. Click to write Item 3 (3)

Q32 If you had the opportunity to respond to a question online, what would you be inclined to? Choose as many that apply

- ☐ I would never respond to online question. If so kindly explain why (1) ____
- ☐ I would answer for financial renumerations (2)
- ☐ Empathic response (3)
- ☐ Short/simplified responses (4)
- ☐ Detailed responses (5)
- ☐ Always ask the inquirer to see a health professional (6)
- ☐ Accuracy (8)
- ☐ Other (7) ____
